# Enhancing prime editing by Csy4-mediated processing of pegRNA

**DOI:** 10.1038/s41422-021-00520-x

**Published:** 2021-06-08

**Authors:** Yao Liu, Guang Yang, Shuhong Huang, Xiangyang Li, Xin Wang, Guanglei Li, Tian Chi, Yulin Chen, Xingxu Huang, Xiaolong Wang

**Affiliations:** 1 Key Laboratory of Animal Genetics, Breeding and Reproduction of Shaanxi Province, College of Animal Science and Technology, Northwest A&F University, Yangling, Shaanxi China; 2 School of Life Science and Technology, ShanghaiTech University, Shanghai, China; 3 CAS Center for Excellence in Molecular Cell Science, Shanghai Institute of Biochemistry and Cell Biology, Chinese Academy of Sciences, Shanghai, China

**Keywords:** DNA recombination, Homologous recombination

Dear Editor,

The most advanced prime editor 3 (PE3) system comprises the editor, a fusion protein of Cas9 H840A nickase and mutant reverse transcriptase (RTase) (hereafter termed NMRT), a prime editing guide RNA (pegRNA) and an alternative single-guide RNA (sgRNA).^1^ The pegRNA contains a primer binding site (PBS) and a reverse transcription (RT) template for introducing new genetic information^1^ (Fig. 1a; Supplementary information, Fig. S1a). We noted that the PBS, which is generally 10–16 nt at the 3′ end of pegRNA, is complementary to part of the spacer at the 5′ end of pegRNA, and their annealing is expected to cause pegRNA circularization, which can potentially hamper editing (Fig. 1a; Supplementary information, Fig. S1b, c).

**Fig. 1:**
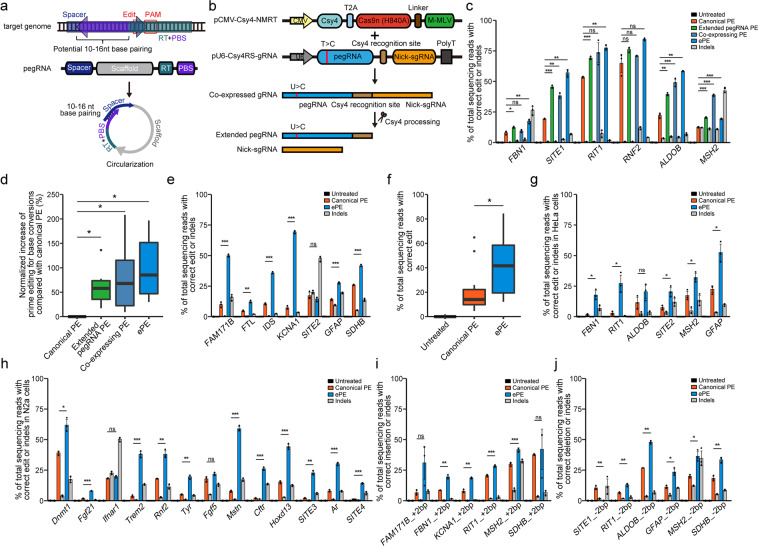
Enhanced prime editing system using Csy4-processed pegRNA. **a** A schematic representation of the circularization formed by the PBS and spacer. A canonical pegRNA consists of spacer, scaffold, RT, and PBS. pegRNA spacer is highlighted in dark blue, scaffold in gray, RT in cyan, PBS in purple. The spacer and the PBS share a complementary sequence, and their annealing is expected to cause pegRNA circularization. **b** ePE system. Csy4 protein is fused to and co-expressed with the prime editor NMRT. pegRNA and nick-sgRNA are fused and co-expressed in a single transcript from a U6 promoter with pegRNA flanked by Csy4 recognition site (Csy4RS). Csy4 nuclease cleaves and releases pegRNA and nick-sgRNA from the transcript. With Csy4 processing, the hairpin Csy4 recognition site remains at the 3′ end of the pegRNA to become extended pegRNA. Mutation of the fourth uracil of consecutive uracils (marked by red line) was introduced to the scaffold of pegRNA. **c** Increasing targeted efficiency of base transition and transversion by extended pegRNA, co-expressing extended pegRNA and nick-sgRNA, and ePE system in HEK293T cells. PCR amplicons from the target regions were analyzed by targeted deep sequencing. The reads only harboring correct edit were counted to evaluate the editing efficiency, and the reads harboring any undesired insertion or deletion were counted to evaluate the indel frequency. Gray bar indicates the indel frequency coupled with the editing efficiency indicated by the left closest bar. **d** Statistical analysis of normalized increase of targeted base transition and transversion editing efficiencies in **c**. **e** ePE system increases targeted efficiency of base transition and transversion at more sites in HEK293T cells. Gray bar indicates the indel frequency coupled with the editing efficiency indicated by the left closest bar. **f** Statistical analysis of prime editing point mutation efficiency by canonical PE and ePE system at all human sites used in **c** and **e**. **g** ePE system increases targeted efficiency of base transition and transversion in HeLa cells. Gray bar indicates the indel frequency coupled with the editing efficiency indicated by the left closest bar. **h** ePE system increases targeted efficiency of base transition and transversion in murine N2a cells. Gray bar indicates the indel frequency coupled with the editing efficiency indicated by the left closest bar. **i** ePE system increases the efficiency of targeted precise sequence insertion in HEK293T cells. Gray bar indicates the indel frequency coupled with the editing efficiency indicated by the left closest bar. **j** ePE system increases the efficiency of targeted precise sequence deletion in HEK293T cells. Gray bar indicates the indel frequency coupled with the editing efficiency indicated by the left closest bar. For **c**–**j**, data are presented as mean values ± SD, *n* = 3 independent experiments, two-tailed Student’s *t*-test (*P < 0.05, **P < 0.005, ***P < 0.0005).

To test this hypothesis, we made non-circularizable derivatives of canonical pegRNA by deleting PBS and RT (“Truncated pegRNA”) or by replacing PBS with a random sequence of the same size as PBS (“RaPBS-pegRNA”) (Supplementary information, Fig. S2a), and then compared their abilities to induce Cas9-mediated DNA indels together with canonical pegRNA at four target genes (*FBN1*, *ALDOB, SITE1*, and *FTL*). Indeed, these two kinds of changes prevented the potential circularization of pegRNA, with the efficiencies of indel induction for canonical pegRNA, truncated pegRNA and RaPBS-pegRNA being 14.2%, 53.2% and 36.0% (at *FBN1)* or 55.4%, 75.5% and 81.4% (at *ALDOB*) or 6.0%, 55.8% and 42.5% (at *SITE1*) or 14.7%, 25.6% and 24.2% (at *FTL*) (Supplementary information, Fig. S2b). We next sought to prevent pegRNA circularization while maintaining the integrity of PBS and RT template, which is essential for pegRNA function in the PE system. To this end, we fused the 20-nt Csy4 recognition site to the 3′ end of canonical pegRNA. This site, naturally present at Type I-F CRISPR-Cas systems,^2^ forms a hairpin,^3^ which might inhibit circularization when appended to the pegRNA. Indeed, the extended pegRNA outperformed the canonical pegRNA in inducing Cas9-meidated indels, increasing the efficiencies from 14.2% to 23.8% at *FBN1*, 55.4% to 74.9% at *ALDOB*, 6.0% to 32.2% at *SITE1* and 14.7% to 23.8% at *FTL*, respectively (Supplementary information, Figs. S2b, c, S3).

Using PE3, we next compared the performance of the canonical pegRNA (canonical PE) and the extended pegRNA (extended pegRNA PE) in generating point mutations, and found that significant increase in targeted base conversion of different editing types at 6 sites tested (Fig. 1c, d; Supplementary information, Fig. S4). We then introduced two more modifications to the extended pegRNA. First, we fused nick-sgRNA to the extended pegRNA, enabling their co-expression in a single transcript, which might help optimize the stoichiometry of the two guides. Meanwhile, to release the nick-sgRNA from the transcript, we fused NMRT with Csy4-T2A, the Csy4 RNase that selectively cleaves at the 3′ end of the Csy4 recognition site.^3^ With pCMV-Csy4-NMRT, expressing the single transcript containing extended pegRNA and nick-sgRNA (the PE is named co-expressing PE) showed an average of 0.8× increase in point mutations at 6 tested sites compared with the canonical PE (Fig. 1c, d; Supplementary information, Fig. S4). The second modification is based on our recent finding that mutating the fourth uracil of consecutive uracils in the scaffold of pegRNA into cytosine eliminated a putative transcription termination signal,^4^ thus increasing the pegRNA expression and prime editing (unpublished data). We thus mutated the fourth uracil of consecutive uracils to cytosine in the scaffold of extended pegRNA. The resultant PE system is termed enhanced prime editing system (ePE) (Fig. 1b; Supplementary information, Figs. S5, S6).

ePE showed an average of 1.0× increase in point mutations at 6 tested sites (Fig. 1c, d; Supplementary information, Fig. S4) and a 2.6× increase at additional sites (Fig. 1e; Supplementary information, Fig. S7) in HEK293T cells. Thus, ePE caused an average of 1.9× increase of editing efficiency for point mutations, compared with the canonical PE (Fig. 1f). Similarly, ePE was 3.8× more active than canonical PE in HeLa cells (Fig. 1g; Supplementary information, Fig. S8) and 4.9× more active in murine N2a cells (Fig. 1h; Supplementary information, Fig. S9). Note that ePE in HeLa cells was less active than in HEK293T cells (Fig. 1c, e, g), consistent with a previous study.^1^ In addition, without nicking the unedited strand, the editing efficiency was significantly lower than that with nick-sgRNA. But ePE still outperformed canonical PE (by 1.9×; Supplementary information, Fig. S10). Furthermore, the length of RT templates did affect the editing efficiencies, but ePE outperformed the canonical PE with RT templates at various lengths at all tested sites except *MSH2* (Supplementary information, Fig. S11). Also, an important application of prime editing is to engender precise insertion and deletion, for which ePE also clearly outperformed canonical PE (1.2× and 0.6× increases, respectively) (Fig. 1i, j; Supplementary information, Fig. S12a, b).

The fidelity of genome editing is of great importance for its therapeutic and clinical application. Three lines of evidence indicate that the fidelity of ePE was comparable to the canonical PE. First, editing byproducts surrounding 2 bp of the target bases were undetectable for ePE as in the case of the canonical PE at all 13 tested human sites (Supplementary information, Fig. S13a). Second, when installing point mutations, ePE induced unintended indels at the targeted sites only slightly more frequently than the canonical PE (*P* = 0.03) (Supplementary information, Fig. S13b). Third, ePE induced similar levels of unintended indels during indel editing (*P* = 0.28 for insertion editing and *P* = 0.17 for deletion editing) (Supplementary information, Fig. S12c, d). Finally, we examined off-target editing by Cas9 nickase at loci predicted by Cas-OFFinder,^5^ finding that the two systems are comparable (Supplementary information, Fig. S14a–c).

In summary, we have introduced multiple modifications into PE to generate ePE, which markedly boosted the editing efficiency. However, ePE may cause slightly more indels and the addition of Csy4 to the system may hamper its delivery. Therefore, further optimization is needed.

## Supplementary information

Supplementary information
