# Supplementary material for: Enhancing prime editing by Csy4-mediated processing of pegRNA
Source: Cell Res. 2021 Jun 8;31(10):1134–6. doi: 10.1038/s41422-021-00520-x (PMC8486859; doi:10.1038/s41422-021-00520-x)
Supplement: Supplementary file 1 — Supplementary information [file 41422_2021_520_MOESM1_ESM.pdf]

SUPPLEMENTARY INFORMATION

Materials and Methods

Plasmid construction

Prime editing system plasmid was purchased from Addgene (Addgene, #132775). pegRNA plasmid was constructed according to the methods described in our previous study[1]. To construct pU6-Csy4RS-gRNA plasmids, the plasmid backbone was amplified from pGL3-U6-sgRNA-EGFP (Addgene, #107721) using Phanta® Max Super Fidelity DNA Polymerase (Vazyme) (Supplementary information, Table S1). The backbone amplicon was then cut by BsaI-HFv2 (NEB) for overhangs. Spacer oligos (the top strand oligo includes 5' ACCG and 3' GTTTC overhangs, while the bottom strand oligo comprises a 5' CTCTGAAAC overhang) were synthesized. pegRNA 3' extension, Csy4 recognition site, and the spacer of nick-sgRNA were synthesized on an oligo (the top strand oligo included 5' GTGC overhang while the bottom strand oligo included 5' AAAC overhang), sgRNA scaffold oligos (featuring compatible overhangs) were synthesized (Supplementary information, Tables S1–S4). The sequence of Csy4 was synthesized by GENEWIZ and cloned to the prime editor using ClonExpress II One Step Cloning Kit (Vazyme, C112-01).

Cell culture, transfection, and harvest

HEK293T, HeLa, and Neuro-2a (N2a) cells were cultured in Dulbecco's Modified Eagle Medium (Gibco) supplemented with 10% fetal bovine serum (FBS) (v/v) (Gemini) and incubated at 37 ℃ with 5% CO_2. For plasmid transfection, cells were seeded on poly-D-lysine-coated 24-well plates and transfected at approximately 70% confluence using EZ Trans (Shanghai Life iLab Biotech Co., Ltd), according to the manufacturer's protocols. A total of 900 ng prime editor, 300 ng pegRNA, and 100 ng corresponding nick sgRNA were transfected into cells per well. 72 h after transfection, GFP+ cells were collected from Fluorescence Activating Cell Sorter (FACS).

Genomic DNA extraction and genotyping

The genomic DNA of GFP+ cells was extracted using QuickExtract™ DNA Extraction Solution (Lucigen) according to manufacturer's protocols. The isolated DNA was PCR-amplified with Phanta® Max Super-Fidelity DNA Polymerase (Vazyme). Primers used are listed in Supplementary information, Table S5.

T7EN1 cleavage assay

Target region was PCR-amplified with Phanta® Max Super-Fidelity DNA Polymerase (Vazyme) from the genomic DNA of GFP+ cells. T7EN1 cleavage assay was performed according to the previous study[2].

Targeted deep-sequencing

Target sites were amplified with Phanta Max Super-Fidelity DNA Polymerase (Vazyme) and subjected to high-throughput sequencing with the Illumina Hiseq X Ten platform. To evaluate the prime editing efficiency, Fastq-multx (V1.3.1) was employed for spliting reads from pool-sequencing, BWA[3] (V0.7.17) and Samtools[4] (V1.7) were employed to map the paired-end reads, and the CRIPResso2 (V2.0.43)[5] was used to analyze the amplicons. Five prime editing info parameters were provided for CRIPResso2 which included pegRNA_spacer_seq, pegRNA_extension_seq, pegRNA_scaffold_seq, nicking_guide_seq and ref_seq, and the other parameters required were set default. The reads only harboring correct edit were counted to evaluate the editing efficiency, and the reads harboring any undesired insertion or deletion were counted to evaluate the indel frequency.

Off-target analysis

Potential off-target sites were predicted in the human genome (GRCh38/hg38) with Cas-OFFinder[6] (http://www.rgenome.net/cas-offinder); The region around the off-target sites were amplified with Phanta Max Super-Fidelity DNA Polymerase (Vazyme), and subjected to high-throughput sequencing with the Illumina Hiseq X Ten platform. The amplicons were analyzed with CRIPResso2 (V2.0.43) and the off-target sites are listed in Supplementary information, Table S6. Primers used are listed in Supplementary information, Table S7.

Data analysis

All data were calculated based on three independent experiments, and Student's *t*-test (two-tailed) was used to calculate the statistical difference. Data are presented as means ± SD.

Data availability

Targeted amplicon sequencing data has been deposited in the NCBI under BioProject number PRJNA687397.

References

1 Liu, Y. *et al.* Efficient generation of mouse models with the prime editing system. *Cell Discov* **6**, 27 (2020).

2 Shen, B. *et al.* Generation of gene-modified mice via Cas9/RNA-mediated gene targeting. *Cell Res* **23**, 720-723 (2013).

3 Li, H. & Durbin, R. Fast and accurate short read alignment with Burrows-Wheeler transform. *Bioinformatics* **25**, 1754-1760 (2009).

4 Li, H. *et al.* The Sequence Alignment/Map format and SAMtools. *Bioinformatics* **25**, 2078-2079 (2009).

5 Clement, K. *et al.* CRISPResso2 provides accurate and rapid genome editing sequence analysis. *Nat Biotechnol* **37**, 224-226 (2019).

6 Bae, S., Park, J. & Kim, J. S. Cas-OFFinder: a fast and versatile algorithm that searches for potential off-target sites of Cas9 RNA-guided endonucleases. *Bioinformatics* **30**, 1473-1475 (2014).

7 Anzalone, A. V. *et al.* Search-and-replace genome editing without double-strand breaks or donor DNA. *Nature* **576**, 149-157 (2019).

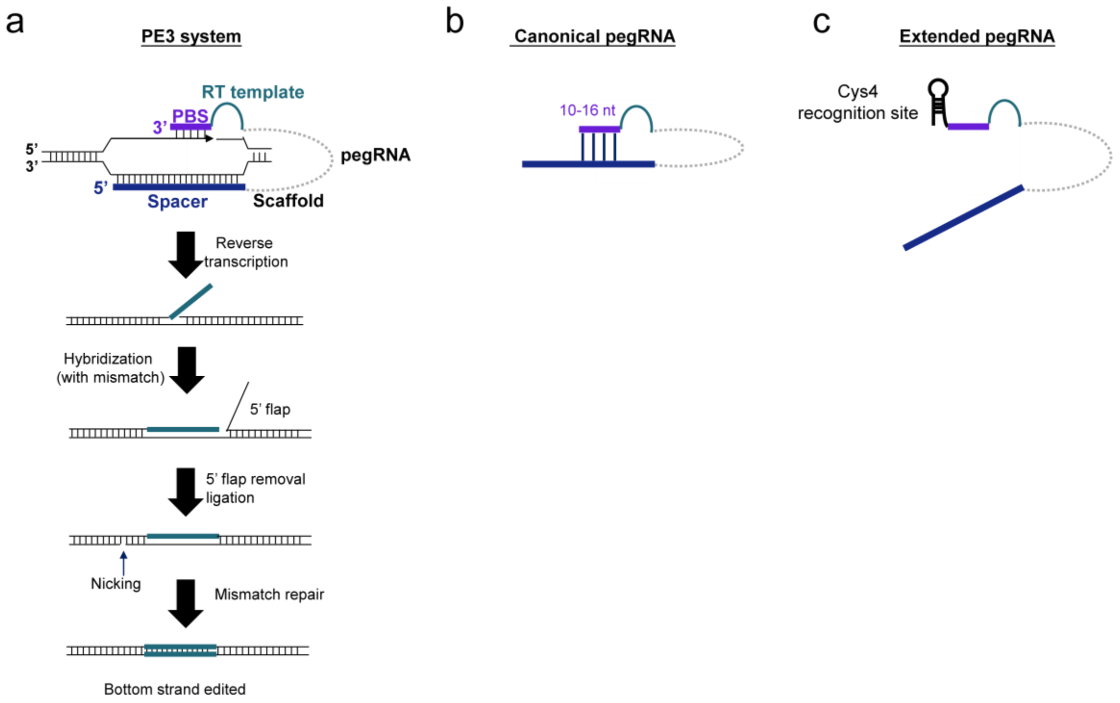

Supplementary information, Fig. S1 Diagrams of PE3 system and pegRNAs.

- **a.** Principle of Prime Editor 3[7]. The first component of the system is pegRNA, which is the same as the conventional Cas9 gRNA except that it additionally carries a reverse transcriptase (RT) template and a primer binding site (PBS) at the 3' end. The pegRNA recruits the second component of the system, a fusion protein consisting of a Cas9 H840A nickase fused to a Moloney Murine Leukemia Virus (M-MLV) reverse transcriptase (not shown). The nickase cleaves the top strand, enabling the sequence upstream of the nick to serve as primer (arrow) for reverse transcription of the RT template. The cDNA then is incorporated into the top strand, and the bottom strand is subsequently nicked to repair the mismatch, copying the edit into the bottom strand in the process.
- **b.** The PBS which is generally 10-16 nt at the 3' end of pegRNA is complementary to the spacer at its 5' end. PBS can potentially bind and sequester the spacer, thus hampering editing.
- **c.** A potential strategy for countering PBS-spacer interaction. The extended pegRNA was generated by fusing a hairpin Csy4 recognition site to the 3' end of canonical pegRNA. Csy4 recognition site may prevent PBS from binding the spacer.

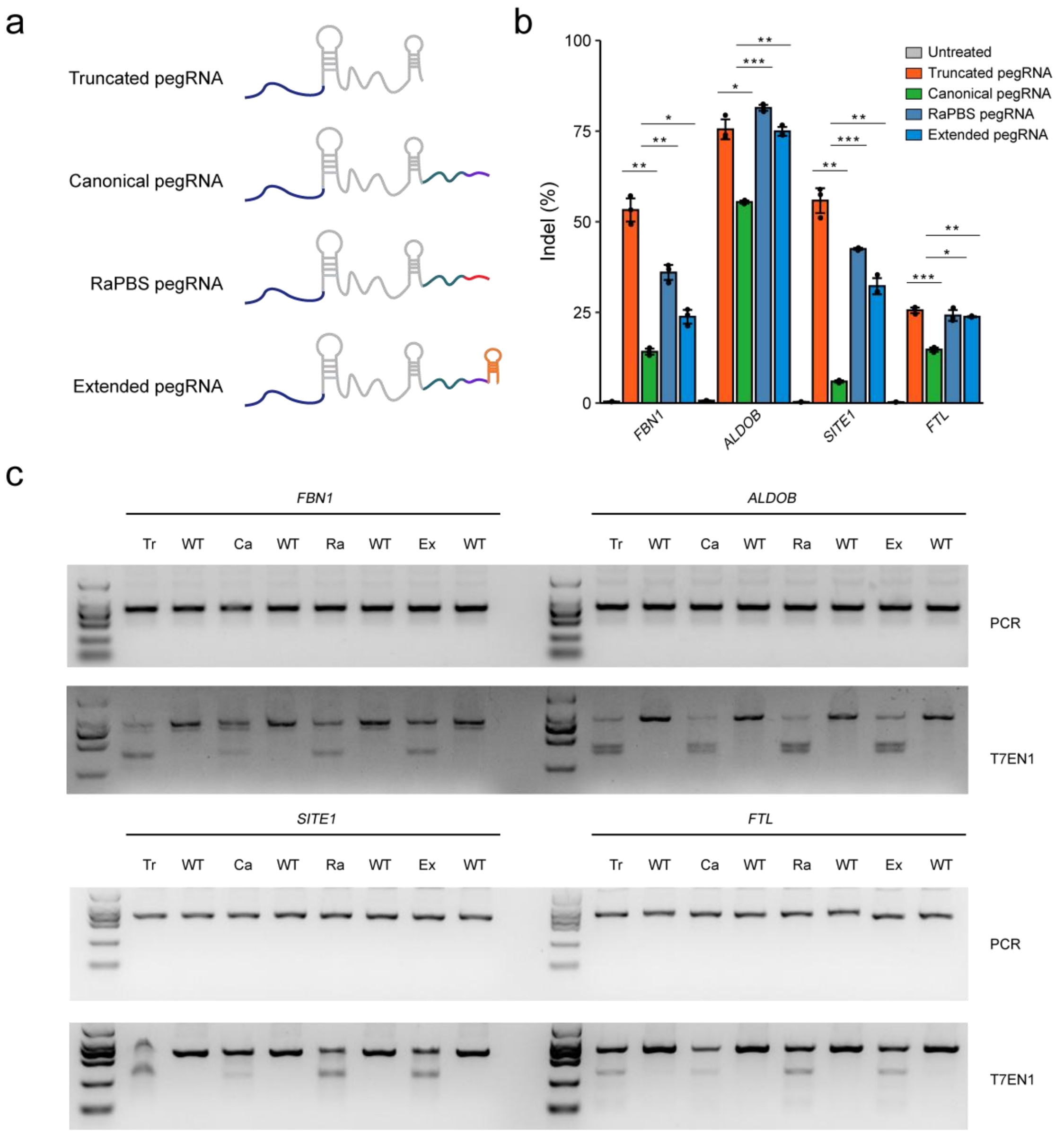

Supplementary information, Fig. S2 Potential circularization of pegRNA weakens the pegRNA activity.

a. The schematic image of differently engineered pegRNAs used for examining DNA cleavage by Cas9 with pegRNA. Truncated pegRNA (without PBS nor RT, same as a typically sgRNA), canonical pegRNA, RaPBS pegRNA (original PBS was replaced by random PBS of same size) and extended pegRNA are shown. Spacer of pegRNA was highlighted in dark blue, scaffold in grey, RT in cyan, PBS in purple, replaced PBS in red, and extended sequence, harpin Csy4 recognition site in orange.

b. Indels induced by different pegRNAs at 4 distinct sites in HEK293T cells. Indels was induced by Cas9 with different engineered pegRNA showed in **(a)** at *FBN1*, *ALDOB*, *SITE1* and *FTL* sites in

HEK293T cells. PCR amplicons from the target regions were analyzed by targeted deep sequencing. Mean values ± SD, n=3 independent experiments (*P < 0.05, **P < 0.005, ***P < 0.0005).

c. T7EN1 cleavage assay were used to evaluate the targeted indels of *FBN1*, *ALDOB*, *SITE1* and *FTL*.

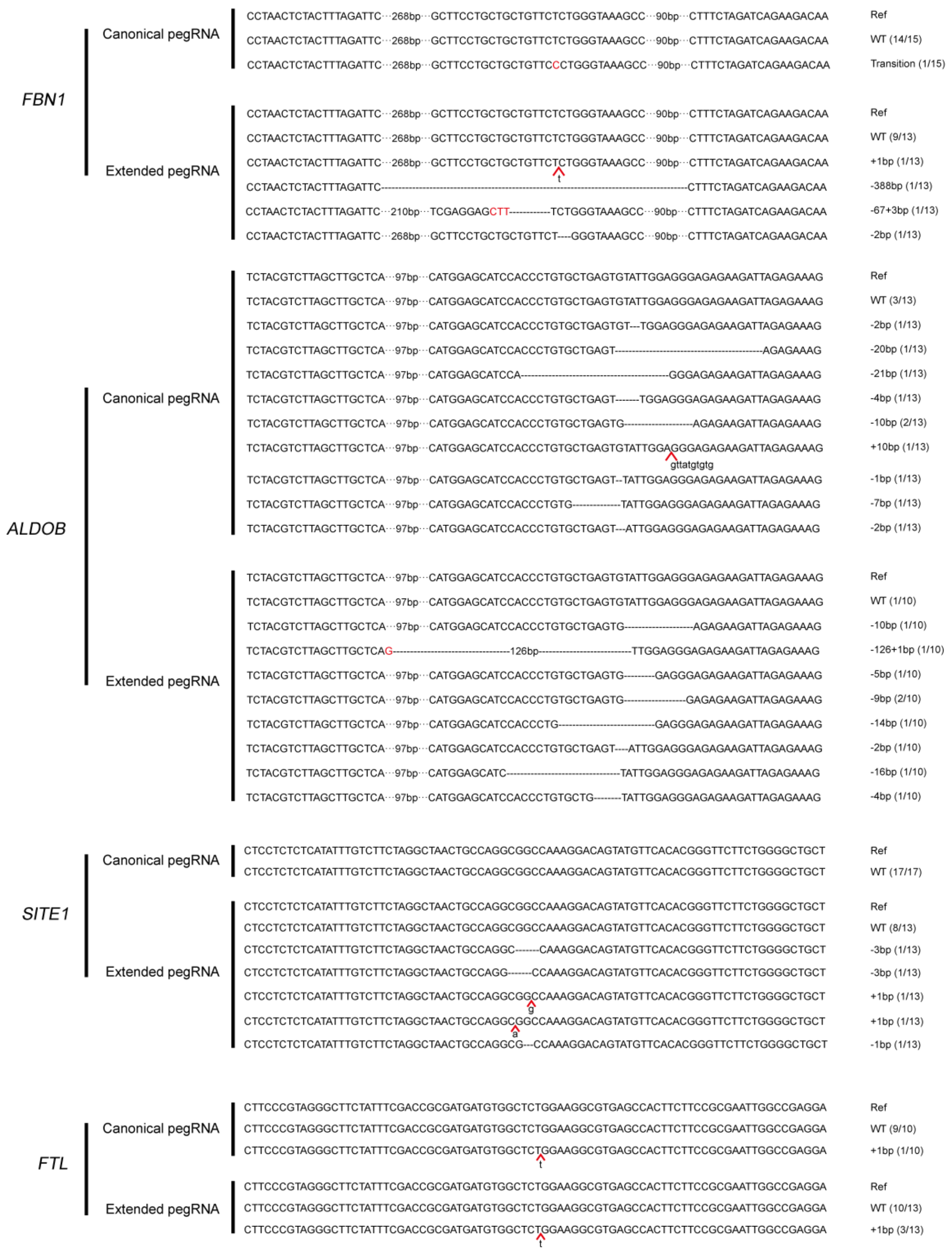

Supplementary information, Fig. S3 T-A clones confirmed the discrepancy of indels induced by Cas9 with extended pegRNA and canonical pegRNA.

T-A clones were used to evaluate indels induced by Cas9 with canonical pegRNAs and extended pegRNAs at *FBN1*, *ALDOB*, *SITE1* and *FTL* sites. N/N indicates the number of colonies with indels out of the number of total samples.

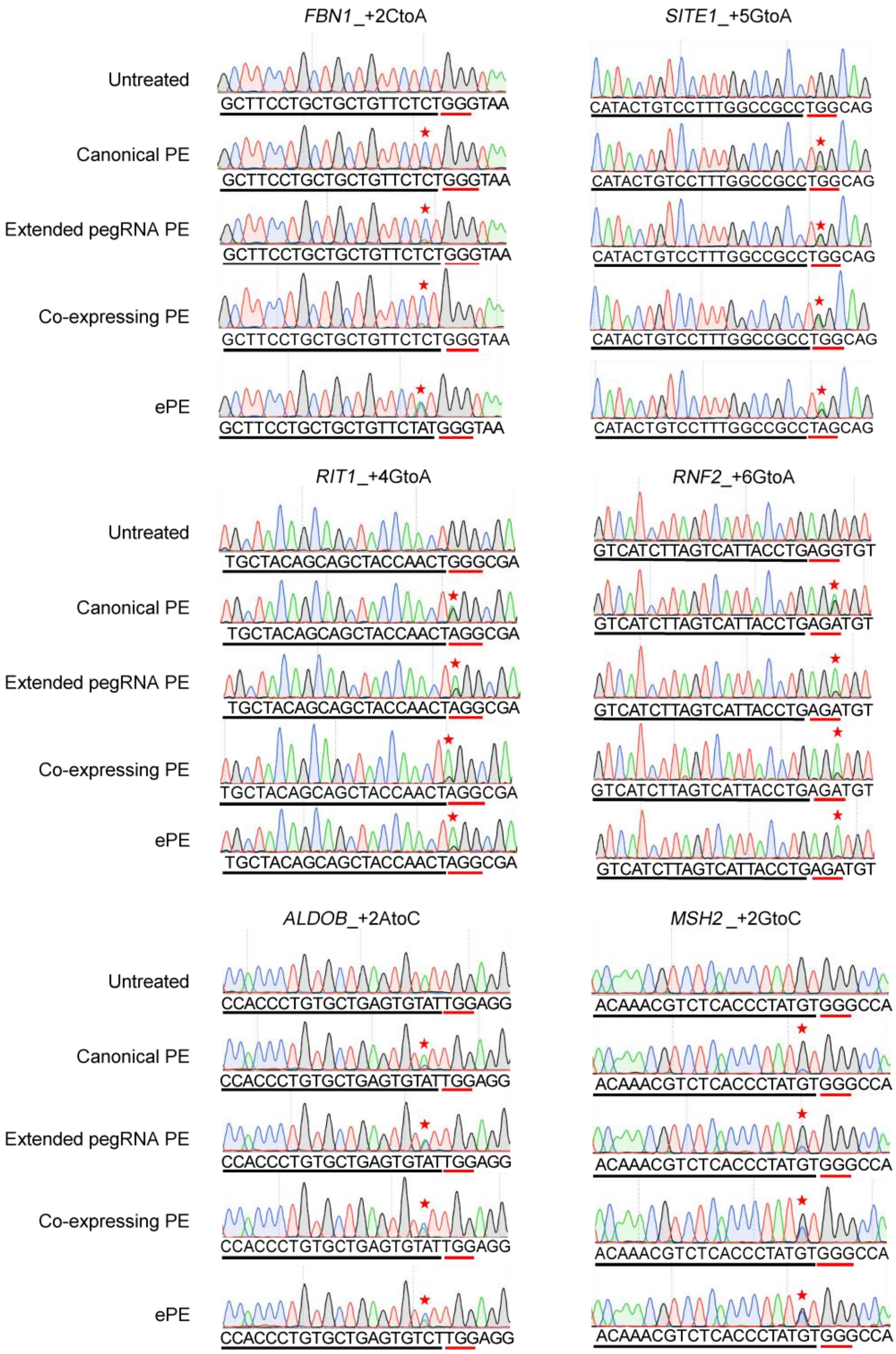

Supplementary information, Fig. S4 Base transition and transversion induced by canonical PE, extended pegRNA PE, co-expressing PE and ePE in HEK293T cells.

Sanger sequencing chromatograms of the six sites in **Fig. 1c**. Asterisks indicate the desired editing.

The PAM sequence and spacer sequence of pegRNA are underlined in red and black, respectively.

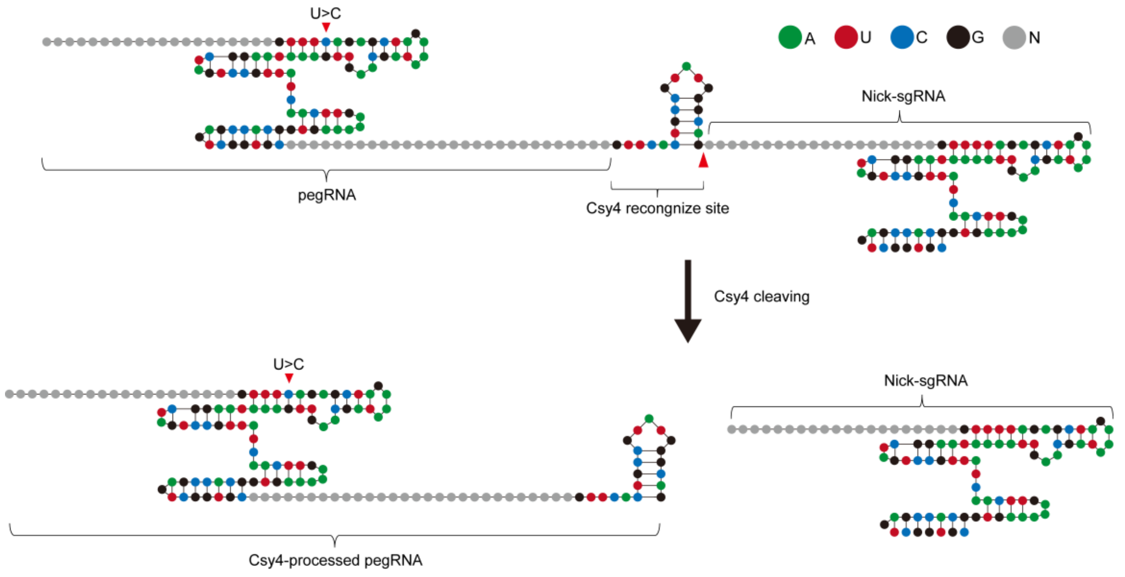

Supplementary information, Fig. S5 RNA sequences of co-expressed pegRNA and nick-sgRNA.

Co-expressed extended pegRNA and nick-sgRNA. Red triangle indicates the cleave site of Csy4 nuclease.

Csy4 nuclease cleaves and releases Csy4-processed pegRNA and nick-sgRNA.

pCMV-Csy4-NMRT coding sequence

(Csy4 colored in red, T2A peptide colored in grey, NLS colored in green, linker colored in purple, Cas9 H840A colored in blue, RTase colored yellow)

ATGGACCACTACCTCGACATTCGCTTGCGACCGGACCCGGAATTTCCCCCGGCGCAACTCATGAGCGTGCTCTTC
GGCAAGCTCCACCAGGCCCTGGTGGCACAGGGCGGGGACAGGATCGGCGTGAGCTTCCCCGACCTCGACGAA
AGCCGCTCCCGGCTGGGCGAGCGCCTGCGCATTCATGCCTCGGCGGACGACCTTCGTGCCCTGCTCGCCCGGC
CCTGGCTGGAAGGGTTGCGGGACCATCTGCAATTCGGAGAACCGGCAGTCGTGCCTCACCCCACACCGTACCGT
CAGGTCAGTCGGGTTCAGGCGAAAAGCAATCCGGAACGCCTGCGGCGGCGGCTCATGCGCCGGCACGATCTGA
GTGAGGAGGAGGCTCGGAAACGCATTCCCGATACGGTCGCGAGAGCCTTGGACCTGCCCTTCGTCACGCTACGC
AGCCAGAGCACCGGACAGCACTTCCGTCTCTTCATCCGCCACGGGCCGTTGCAGGTGACGGCAGAGGAAGGAG
GATTCACCTGTTACGGGTTGAGCAAAGGAGGTTTCGTTCCCTGGTTCGAGGGCAGAGGAAGTCTGTTAACATGCG
GTGACGTCGAGGAGAATCCTGGCCCACCAAAGAAGAAGCGGAAAGTCGACAAGAAGTACAGCATCGGCCTGGAC
ATCGGCACCAACTCTGTGGGCTGGGCCGTGATCACCGACGAGTACAAGGTGCCCAGCAAGAAATTCAAGGTGCT
GGGCAACACCGACCGGCACAGCATCAAGAAGAACCTGATCGGAGCCCTGCTGTTCGACAGCGGCGAAACAGCC
GAGGCCACCCGGCTGAAGAGAACCGCCAGAAGAAGATACACCAGACGGAAGAACCGGATCTGCTATCTGCAAGA
GATCTTCAGCAACGAGATGGCCAAGGTGGACGACAGCTTCTTCCACAGACTGGAAGAGTCCTTCCTGGTGGAAG
AGGATAAGAAGCACGAGCGGCACCCCATCTTCGGCAACATCGTGGACGAGGTGGCCTACCACGAGAAGTACCCC
ACCATCTACCACCTGAGAAAGAAACTGGTGGACAGCACCGACAAGGCCGACCTGCGGCTGATCTATCTGGCCCT
GGCCCACATGATCAAGTTCCGGGGCCACTTCCTGATCGAGGGCGACCTGAACCCCGACAACAGCGACGTGGACA
AGCTGTTCATCCAGCTGGTGCAGACCTACAACCAGCTGTTCGAGGAAAACCCCATCAACGCCAGCGGCGTGGAC
GCCAAGGCCATCCTGTCTGCCAGACTGAGCAAGAGCAGACGGCTGGAAAATCTGATCGCCCAGCTGCCCGGCG
AGAAGAAGAATGGCCTGTTCGGAAACCTGATTGCCCTGAGCCTGGGCCTGACCCCCAACTTCAAGAGCAACTTC
GACCTGGCCGAGGATGCCAAACTGCAGCTGAGCAAGGACACCTACGACGACGACCTGGACAACCTGCTGGCCC
AGATCGGCGACCAGTACGCCGACCTGTTTCTGGCCGCCAAGAACCTGTCCGACGCCATCCTGCTGAGCGACATC
CTGAGAGTGAACACCGAGATCACCAAGGCCCCCCTGAGCGCCTCTATGATCAAGAGATACGACGAGCACCACCA
GGACCTGACCCTGCTGAAAGCTCTCGTGCGGCAGCAGCTGCCTGAGAAGTACAAAGAGATTTTCTTCGACCAGA
GCAAGAACGGCTACGCCGGCTACATTGACGGCGGAGCCAGCCAGGAAGAGTTCTACAAGTTCATCAAGCCCATC
CTGGAAAAGATGGACGGCACCGAGGAACTGCTCGTGAAGCTGAACAGAGAGGACCTGCTGCGGAAGCAGCGGA
CCTTCGACAACGGCAGCATCCCCCACCAGATCCACCTGGGAGAGCTGCACGCCATTCTGCGGCGGCAGGAAGAT
TTTTACCCATTCCTGAAGGACAACCGGGAAAAGATCGAGAAGATCCTGACCTTCCGCATCCCCTACTACGTGGGC
CCTCTGGCCAGGGGAAACAGCAGATTCGCCTGGATGACCAGAAAGAGCGAGGAAACCATCACCCCCTGGAACTT
CGAGGAAGTGGTGGACAAGGGCGCTTCCGCCCAGAGCTTCATCGAGCGGATGACCAACTTCGATAAGAACCTGC
CCAACGAGAAGGTGCTGCCCAAGCACAGCCTGCTGTACGAGTACTTCACCGTGTATAACGAGCTGACCAAAGTGA
AATACGTGACCGAGGGAATGAGAAAGCCCGCCTTCCTGAGCGGCGAGCAGAAAAAGGCCATCGTGGACCTGCTG
TTCAAGACCAACCGGAAAGTGACCGTGAAGCAGCTGAAAGAGGACTACTTCAAGAAAATCGAGTGCTTCGACTCC
GTGGAAATCTCCGGCGTGGAAGATCGGTTCAACGCCTCCCTGGGCACATACCACGATCTGCTGAAAATTATCAAG
GACAAGGACTTCCTGGACAATGAGGAAAACGAGGACATTCTGGAAGATATCGTGCTGACCCTGACACTGTTTGAG
GACAGAGAGATGATCGAGGAACGGCTGAAAACCTATGCCCACCTGTTCGACGACAAAGTGATGAAGCAGCTGAA
GCGGCGGAGATACACCGGCTGGGGCAGGCTGAGCCGGAAGCTGATCAACGGCATCCGGGACAAGCAGTCCGG
CAAGACAATCCTGGATTTCCTGAAGTCCGACGGCTTCGCCAACAGAAACTTCATGCAGCTGATCCACGACGACAG
CCTGACCTTTAAAGAGGACATCCAGAAAGCCCAGGTGTCCGGCCAGGGCGATAGCCTGCACGAGCACATTGCCA
ATCTGGCCGGCAGCCCCGCCATTAAGAAGGGCATCCTGCAGACAGTGAAGGTGGTGGACGAGCTCGTGAAAGTG
ATGGGCCGGCACAAGCCCGAGAACATCGTGATCGAAATGGCCAGAGAGAACCAGACCACCCAGAAGGGACAGAA
GAACAGCCGCGAGAGAATGAAGCGGATCGAAGAGGGCATCAAAGAGCTGGGCAGCCAGATCCTGAAAGAACACC
CCGTGGAAAACACCCAGCTGCAGAACGAGAAGCTGTACCTGTACTACCTGCAGAATGGGCGGGATATGTACGTGG

ACCAGGAACTGGACATCAACCGGCTGTCCGACTACGATGTGGACGCTATCGTGCCTCAGAGCTTTCTGAAGGACG
ACTCCATCGACAACAAGGTGCTGACCAGAAGCGACAAGAACCGGGGCAAGAGCGACAACGTGCCCTCCGAAGA
GGTCGTGAAGAAGATGAAGAACTACTGGCGGCAGCTGCTGAACGCCAAGCTGATTACCCAGAGAAAGTTCGACA
ATCTGACCAAGGCCGAGAGAGGCGGCCTGAGCGAACTGGATAAGGCCGGCTTCATCAAGAGACAGCTGGTGGAA
ACCCGGCAGATCACAAAGCACGTGGCACAGATCCTGGACTCCCGGATGAACACTAAGTACGACGAGAATGACAA
GCTGATCCGGGAAGTGAAAGTGATCACCCTGAAGTCCAAGCTGGTGTCCGATTTCCGGAAGGATTTCCAGTTTTA
CAAAGTGCGCGAGATCAACAACTACCACCACGCCCACGACGCCTACCTGAACGCCGTCGTGGGAACCGCCCTGA
TCAAAAAGTACCCTAAGCTGGAAAGCGAGTTCGTGTACGGCGACTACAAGGTGTACGACGTGCGGAAGATGATCG
CCAAGAGCGAGCAGGAAATCGGCAAGGCTACCGCCAAGTACTTCTTCTACAGCAACATCATGAACTTTTTCAAGAC
CGAGATTACCCTGGCCAACGGCGAGATCCGGAAGCGGCCTCTGATCGAGACAAACGGCGAAACCGGGGAGATC
GTGTGGGATAAGGGCCGGGATTTTGCCACCGTGCGGAAAGTGCTGAGCATGCCCCAAGTGAATATCGTGAAAAAG
ACCGAGGTGCAGACAGGCGGCTTCAGCAAAGAGTCTATCCTGCCCAAGAGGAACAGCGATAAGCTGATCGCCAG
AAAGAAGGACTGGGACCCTAAGAAGTACGGCGGCTTCGACAGCCCCACCGTGGCCTATTCTGTGCTGGTGGTGG
CCAAAGTGGAAAAGGGCAAGTCCAAGAAACTGAAGAGTGTGAAAGAGCTGCTGGGGATCACCATCATGGAAAGA
AGCAGCTTCGAGAAGAATCCCATCGACTTTCTGGAAGCCAAGGGCTACAAAGAAGTGAAAAAGGACCTGATCATC
AAGCTGCCTAAGTACTCCCTGTTCGAGCTGGAAAACGGCCGGAAGAGAATGCTGGCCTCTGCCGGCGAACTGCA
GAAGGGAAACGAACTGGCCCTGCCCTCCAAATATGTGAACTTCCTGTACCTGGCCAGCCACTATGAGAAGCTGAA
GGGCTCCCCCGAGGATAATGAGCAGAAACAGCTGTTTGTGGAACAGCACAAGCACTACCTGGACGAGATCATCGA
GCAGATCAGCGAGTTCTCCAAGAGAGTGATCCTGGCCGACGCTAATCTGGACAAAGTGCTGTCCGCCTACAACAA
GCACCGGGATAAGCCCATCAGAGAGCAGGCCGAGAATATCATCCACCTGTTTACCCTGACCAATCTGGGAGCCCC
TGCCGCCTTCAAGTACTTTGACACCACCATCGACCGGAAGAGGTACACCAGCACCAAAGAGGTGCTGGACGCCA
CCCTGATCCACCAGAGCATCACCGGCCTGTACGAGACACGGATCGACCTGTCTCAGCTGGGAGGTGACTCTGGA
GGATCTAGCGGAGGATCCTCTGGCAGCGAGACACCAGGAACAAGCGAGTCAGCAACACCAGAGAGCAGTGGCG
GCAGCAGCGGCGGCAGCAGCACCCTAAATATAGAAGATGAGTATCGGCTACATGAGACCTCAAAAGAGCCAGATG
TTTCTCTAGGGTCCACATGGCTGTCTGATTTTCCTCAGGCCTGGGCGGAAACCGGGGGCATGGGACTGGCAGTT
CGCCAAGCTCCTCTGATCATACCTCTGAAAGCAACCTCTACCCCCGTGTCCATAAAACAATACCCCATGTCACAAG
AAGCCAGACTGGGGATCAAGCCCCACATACAGAGACTGTTGGACCAGGGAATACTGGTACCCTGCCAGTCCCCC
TGGAACACGCCCCTGCTACCCGTTAAGAAACCAGGGACTAATGATTATAGGCCTGTCCAGGATCTGAGAGAAGTC
AACAAGCGGGTGGAAGACATCCACCCCACCGTGCCCAACCCTTACAACCTCTTGAGCGGGCTCCCACCGTCCCA
CCAGTGGTACACTGTGCTTGATTTAAAGGATGCCTTTTTCTGCCTGAGACTCCACCCCACCAGTCAGCCTCTCTTC
GCCTTTGAGTGGAGAGATCCAGAGATGGGAATCTCAGGACAATTGACCTGGACCAGACTCCCACAGGGTTTCAAA
AACAGTCCCACCCTGTTTAATGAGGCACTGCACAGAGACCTAGCAGACTTCCGGATCCAGCACCCAGACTTGATC
CTGCTACAGTACGTGGATGACTTACTGCTGGCCGCCACTTCTGAGCTAGACTGCCAACAAGGTACTCGGGCCCTG
TTACAAACCCTAGGGAACCTCGGGTATCGGGCCTCGGCCAAGAAAGCCCAAATTTGCCAGAAACAGGTCAAGTAT
CTGGGGTATCTTCTAAAAGAGGGTCAGAGATGGCTGACTGAGGCCAGAAAAGAGACTGTGATGGGGCAGCCTAC
TCCGAAGACCCCTCGACAACTAAGGGAGTTCCTAGGGAAGGCAGGCTTCTGTCGCCTCTTCATCCCTGGGTTTGC
AGAAATGGCAGCCCCCCTGTACCCTCTCACCAAACCGGGGACTCTGTTTAATTGGGGCCCAGACCAACAAAAGGC
CTATCAAGAAATCAAGCAAGCTCTTCTAACTGCCCCAGCCCTGGGGTTGCCAGATTTGACTAAGCCCTTTGAACTC
TTTGTCGACGAGAAGCAGGGCTACGCCAAAGGTGTCCTAACGCAAAAACTGGGACCTTGGCGTCGGCCGGTGGC
CTACCTGTCCAAAAAGCTAGACCCAGTAGCAGCTGGGTGGCCCCCTTGCCTACGGATGGTAGCAGCCATTGCCGT
ACTGACAAAGGATGCAGGCAAGCTAACCATGGGACAGCCACTAGTCATTCTGGCCCCCCATGCAGTAGAGGCACT
AGTCAAACAACCCCCCGACCGCTGGCTTTCCAACGCCCGGATGACTCACTATCAGGCCTTGCTTTTGGACACGGA
CCGGGTCCAGTTCGGACCGGTGGTAGCCCTGAACCCGGCTACGCTGCTCCCACTGCCTGAGGAAGGGCTGCAA
CACAACTGCCTTGATATCCTGGCCGAAGCCCACGGAACCCGACCCGACCTAACGGACCAGCCGCTCCCAGACGC
CGACCACACCTGGTACACGGATGGAAGCAGTCTCTTACAAGAGGGACAGCGTAAGGCGGGAGCTGCGGTGACCA
CCGAGACCGAGGTAATCTGGGCTAAAGCCCTGCCAGCCGGGACATCCGCTCAGCGGGCTGAACTGATAGCACTC
ACCCAGGCCCTAAAGATGGCAGAAGGTAAGAAGCTAAATGTTTATACTGATAGCCGTTATGCTTTTGCTACTGCCCA
TATCCATGGAGAAATATACAGAAGGCGTGGGTGGCTCACATCAGAAGGCAAAGAGATCAAAAATAAAGACGAGATC
TTGGCCCTACTAAAAGCCCTCTTTCTGCCCAAAAGACTTAGCATAATCCATTGTCCAGGACATCAAAAGGGACACA

GCGCCGAGGCTAGAGGCAACCGGATGGCTGACCAAGCGGCCCGAAAGGCAGCCATCACAGAGACTCCAGACAC
CTCTACCCTCCTCATAGAAAATTCATCACCCTCTGGCGGCTCAAAAAGAACCGCCGACGGCAGCGAATTCGAGCC
CAAGAAGAAGAGGAAAGTCTAA※

pU6-Csy4RS-gRNA coding sequence

(spacer of pegRNA colored grey, spacer of nick-sgRNA colored in yellow, scaffolds colored in blue, RT+PBS colored in red, Csy4 recognition site colored in purple)

NNNNNNNNNNNNNNNNNNNNGTTTCAGAGCTAGAAATAGCAAGTTGAAATAAGGCTAGTCCGTTATCAACTTGAAA
AAGTGGCACCGAGTCGGTGCNNNNNNNNNNNNNNNNNNNNNNNNNNNNGTTCACTGCCGTATAGGCAGNNNNN
NNNNNNNNNNNNNNNGTTTTAGAGCTAGAAATAGCAAGTTAAAATAAGGCTAGTCCGTTATCAACTTGAAAAAGTG
GCACCGAGTCGGTGC

Supplementary information, Fig. S6 Coding sequences of pCMV-Csy4-NMRT and pU6-Csy4RS-gRNA used in this study

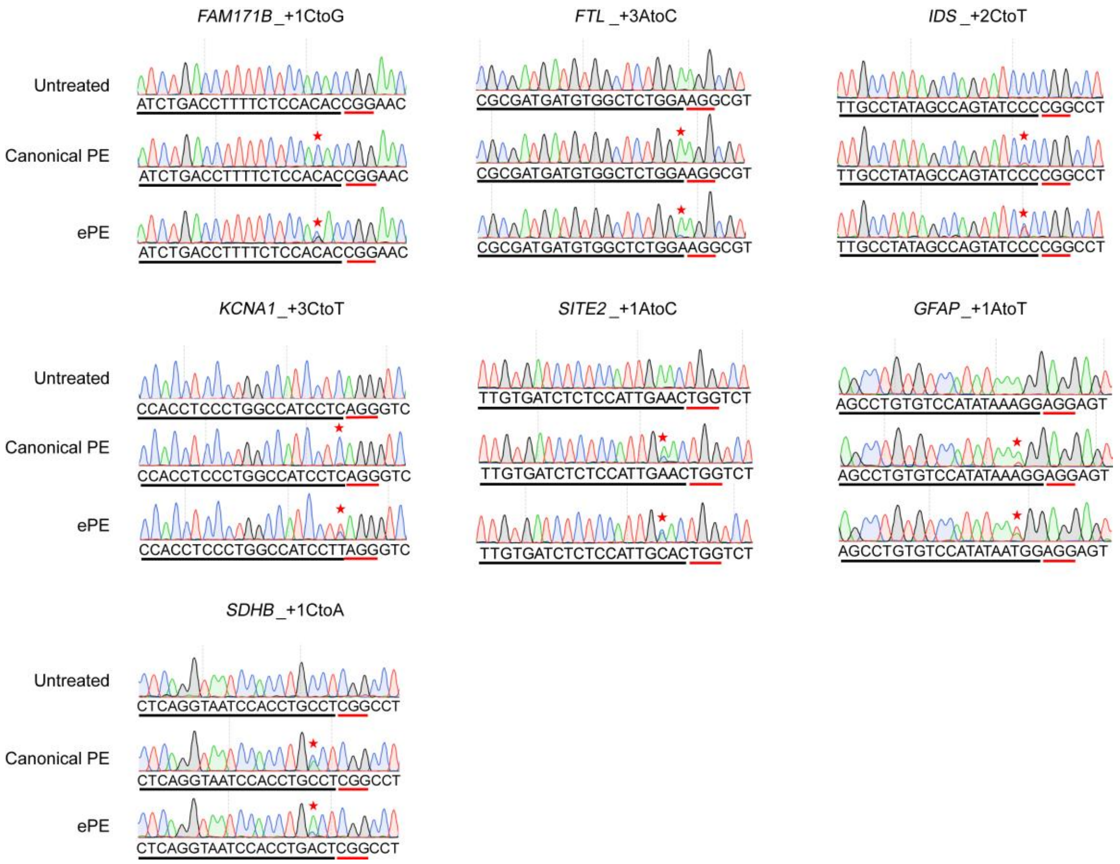

Supplementary information, Fig. S7 Enhancing point mutation efficiency by ePE in HEK293T cells. Sanger sequencing chromatograms of targeted mutations by canonical PE and ePE of 7 sites indicated in **Fig. 1e** in HEK293T cells. Asterisks indicate the desired editing. The PAM sequence and spacer sequence of pegRNA are underlined in red and black, respectively.

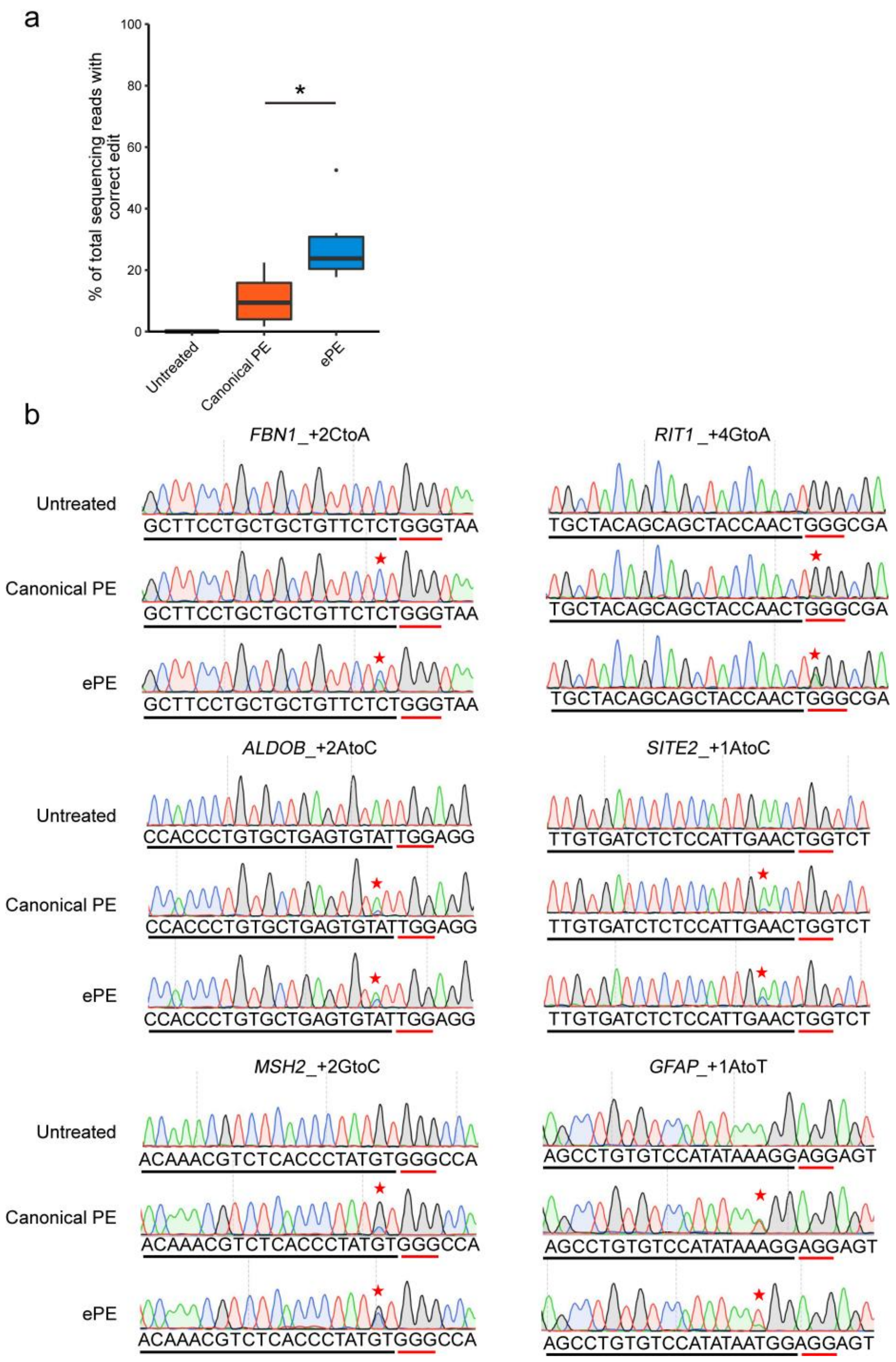

Supplementary information, Fig. S8 ePE increases targeted base transition and transversion efficiency in HeLa cells.

a. Statistical analysis of increase for targeted base transition and transversion in **Fig. 1g**. Data are presented as mean values ± SD, n = 3 independent experiments, two-tailed student's t-test (*$P < 0.05$, **$P < 0.005$, ***$P < 0.0005$).

b. Sanger sequencing chromatograms of the sites in **Fig. 1g**. The PAM sequence and spacer sequence of pegRNA are underlined in red and black, respectively. Asterisks indicate the desired editing.

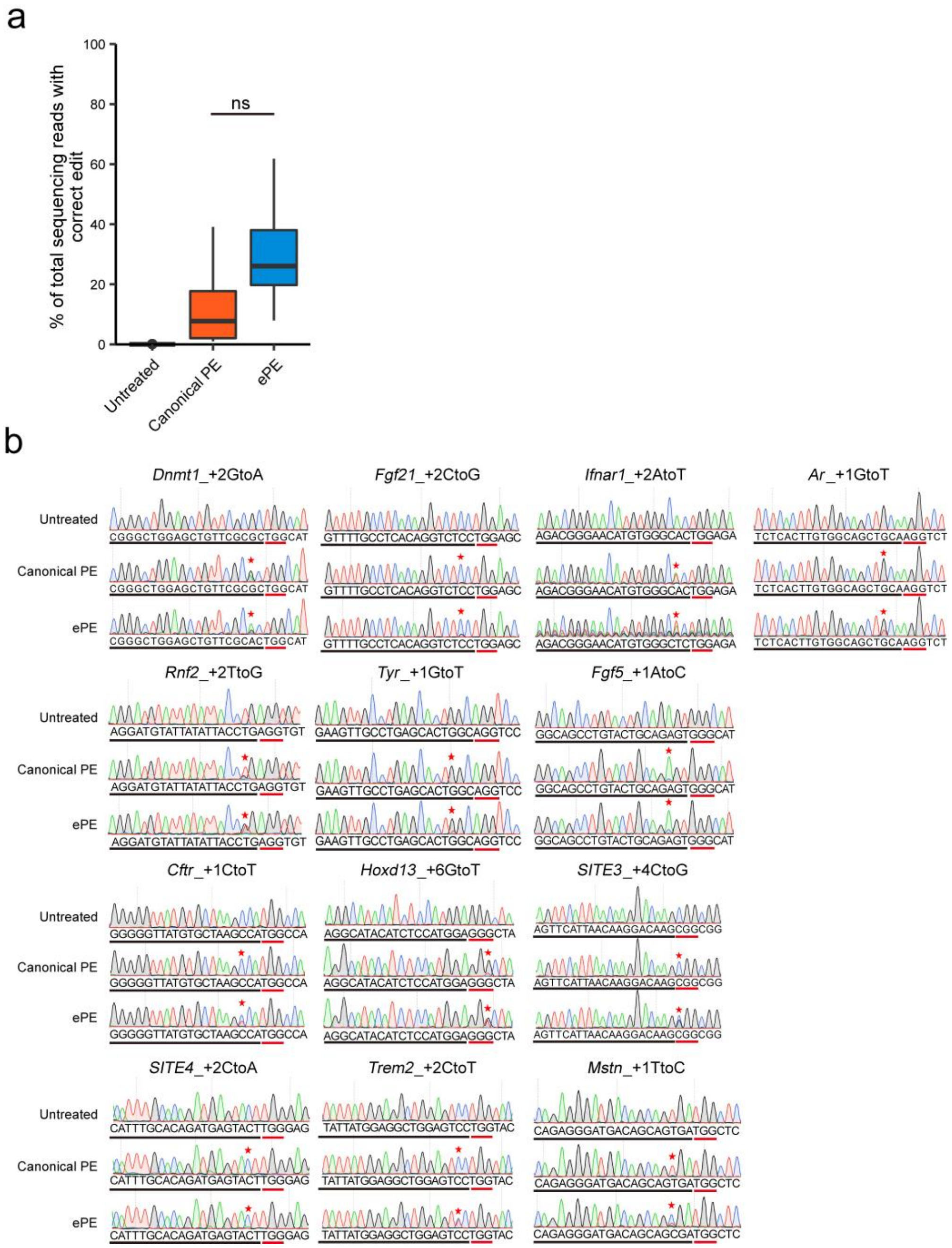

Supplementary information, Fig. S9 Increasing base transition and transversion efficiency by ePE in N2a cells.

a. Statistical analysis of efficiency of targeted base transition and transversion in **Fig. 1h**. Data are presented as mean values ± SD, n = 3 independent experiments, two-tailed student's t-test (*$P < 0.05$, **$P < 0.005$, ***$P < 0.0005$).

b. Sanger sequencing chromatograms of the sites in **Fig. 1h**. The PAM sequence and spacer sequence of pegRNA are underlined in red and black, respectively. Asterisks indicate the desired editing.

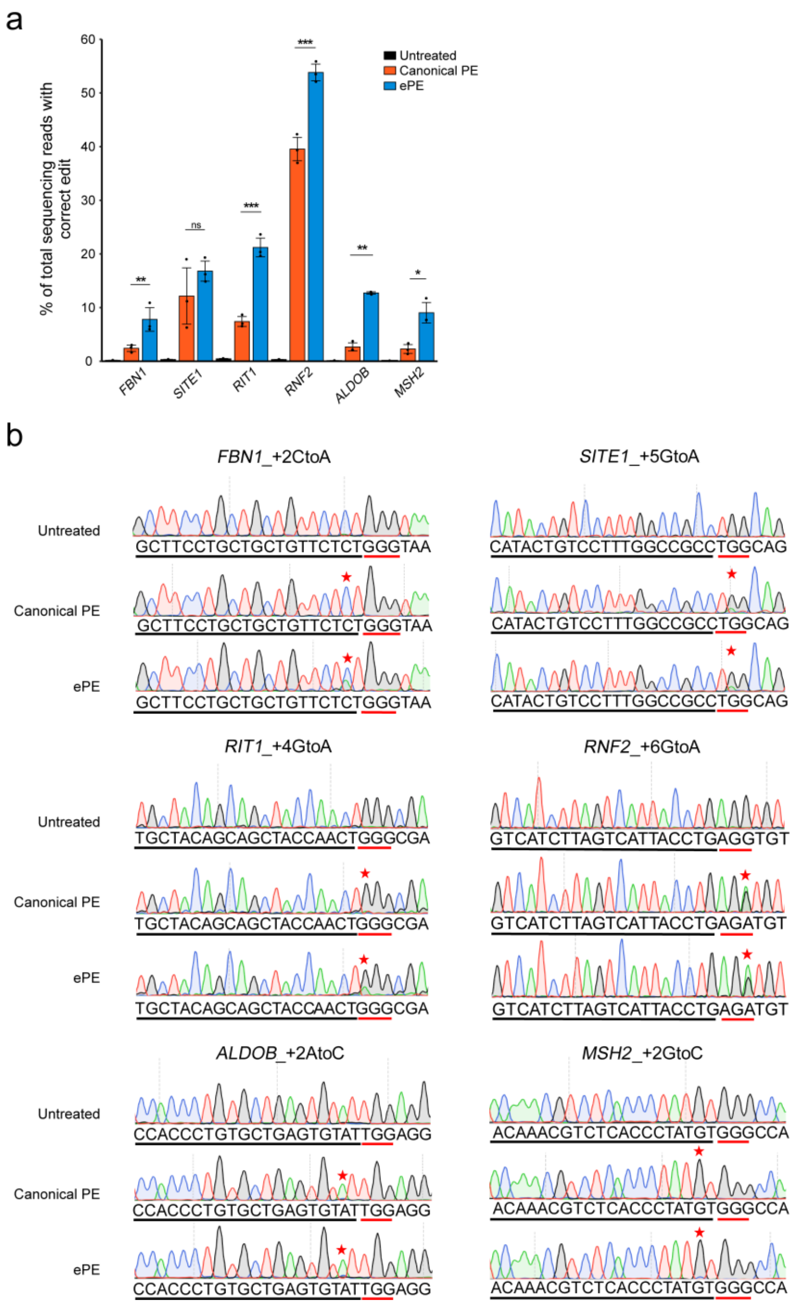

Supplementary information, Fig. S10 ePE without nicking the non-edited strand also increases base transition and transversion efficiency.

a. Targeted editing efficiency of base transition and transversion by canonical PE and ePE without nicking the non-edited strand at six sites in HEK293T cells. Data are presented as mean values ± SD, *n* = 3 independent experiments, two-tailed student's t-test (*P < 0.05, **P < 0.005, ***P < 0.0005).

b. Sanger sequencing chromatograms of the sites in **(a)**. The PAM sequence and spacer sequence of

pegRNA are underlined in red and black, respectively. Asterisks indicate the desired editing.

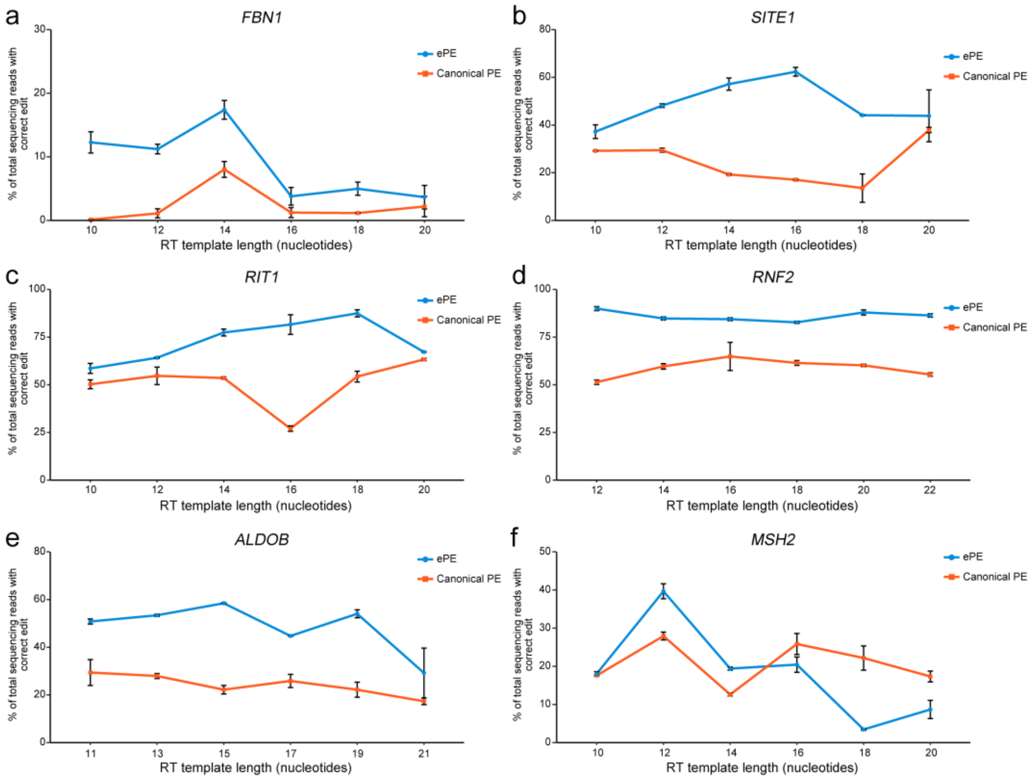

Supplementary information, Fig. S11 Effects of RT template length on the efficiency of ePE in HEK293T cells.

Targeted editing efficiency of base transition and transversion by canonical PE and ePE with different RT template lengths at six sites in HEK293T cells.

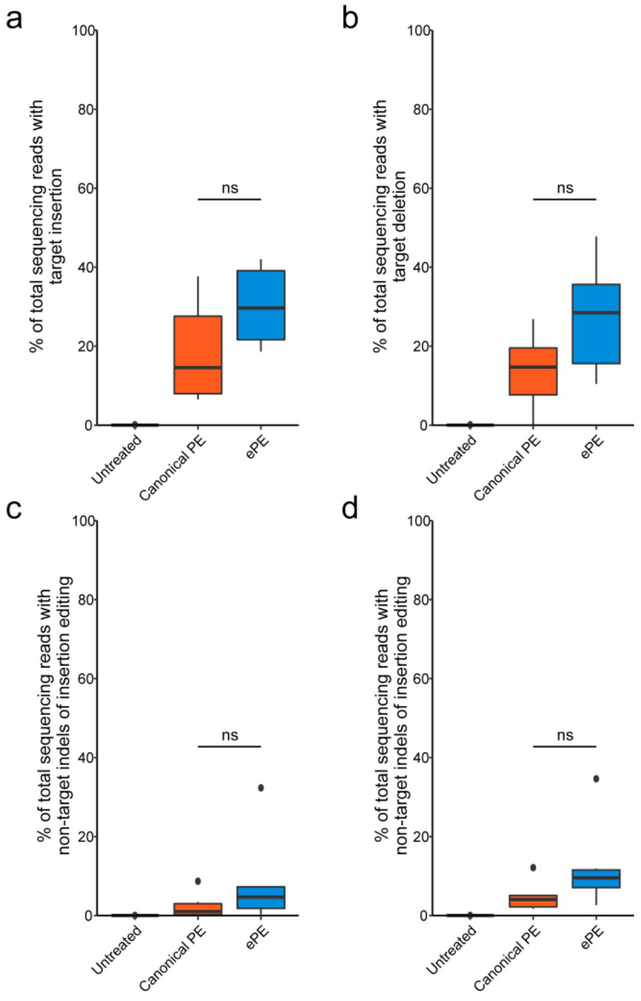

Supplementary information, Fig. S12 ePE increases targeted precise deletion and insertion.

a. Statistical analysis of editing efficiency of targeted precise insertion with canonical PE and ePE in **Fig. 1i**.

b. Statistical analysis of editing efficiency of targeted precise deletion with canonical PE and ePE in **Fig. 1j**.

c. Analysis of potential non-target indels induced by canonical PE and ePE in **Fig. 1i**.

d. Analysis of potential non-target indels induced by canonical PE and ePE in **Fig. 1j**. Mean values ± SD, n = 3 independent experiments, two-tailed student's t-test (*P < 0.05, **P < 0.005, ***P < 0.0005).

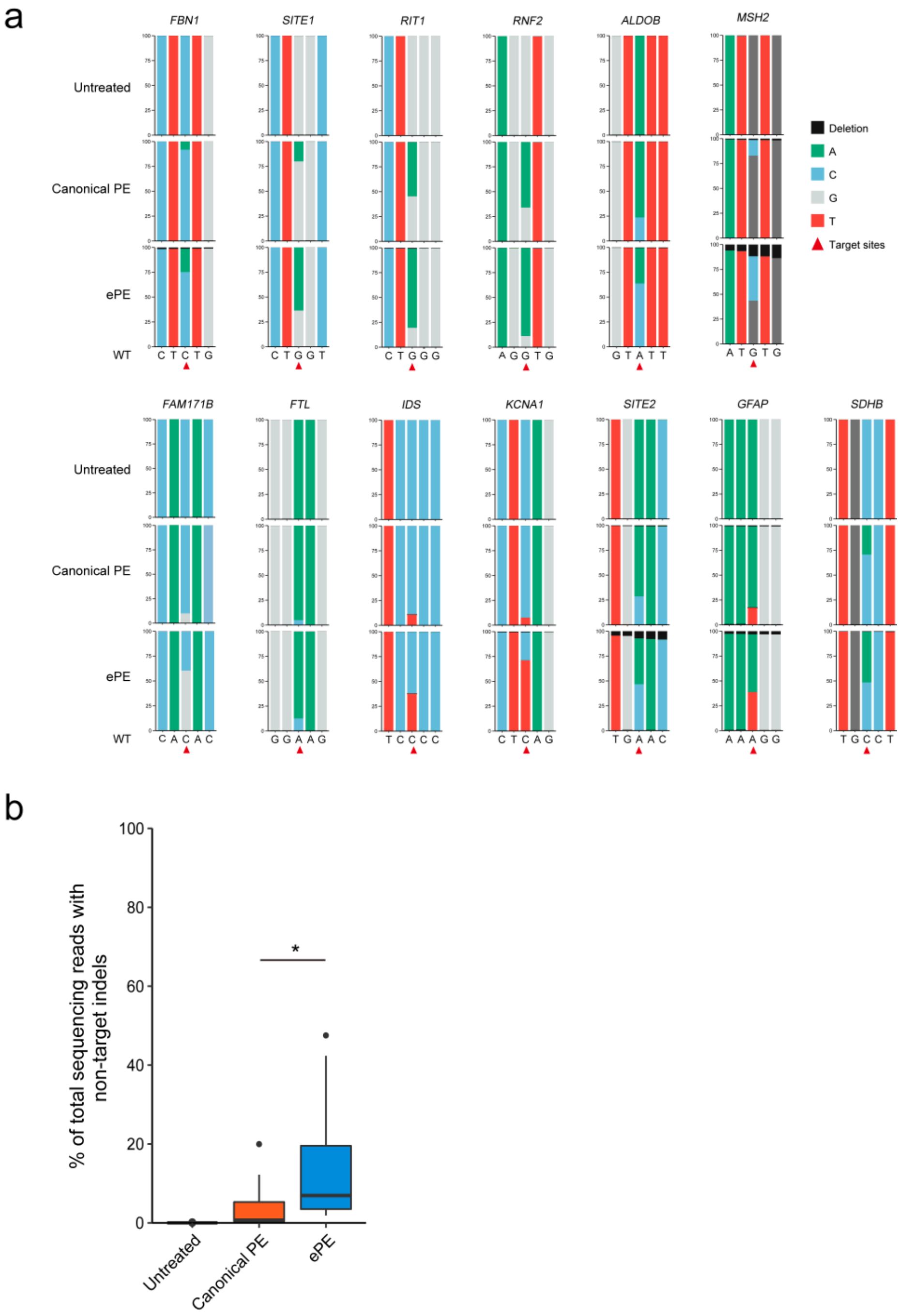

Supplementary information, Fig. S13 Analysis of byproduct and non-target indel induced by canonical PE and ePE.

a. Analysis of byproduct induced by targeted base conversions by canonical PE and EPE for 13 sites in HEK293T cells. Targeted base and nearby 2 bp sequence were shown. The red triangle indicates

the targeted base.

b. Statistical analysis of unintended indels induced by canonical PE and ePE for 13 sites in HEK293T cells. Mean values ± SD, n=3 independent experiments (**P* < 0.05, ***P* < 0.005, ****P* < 0.0005).

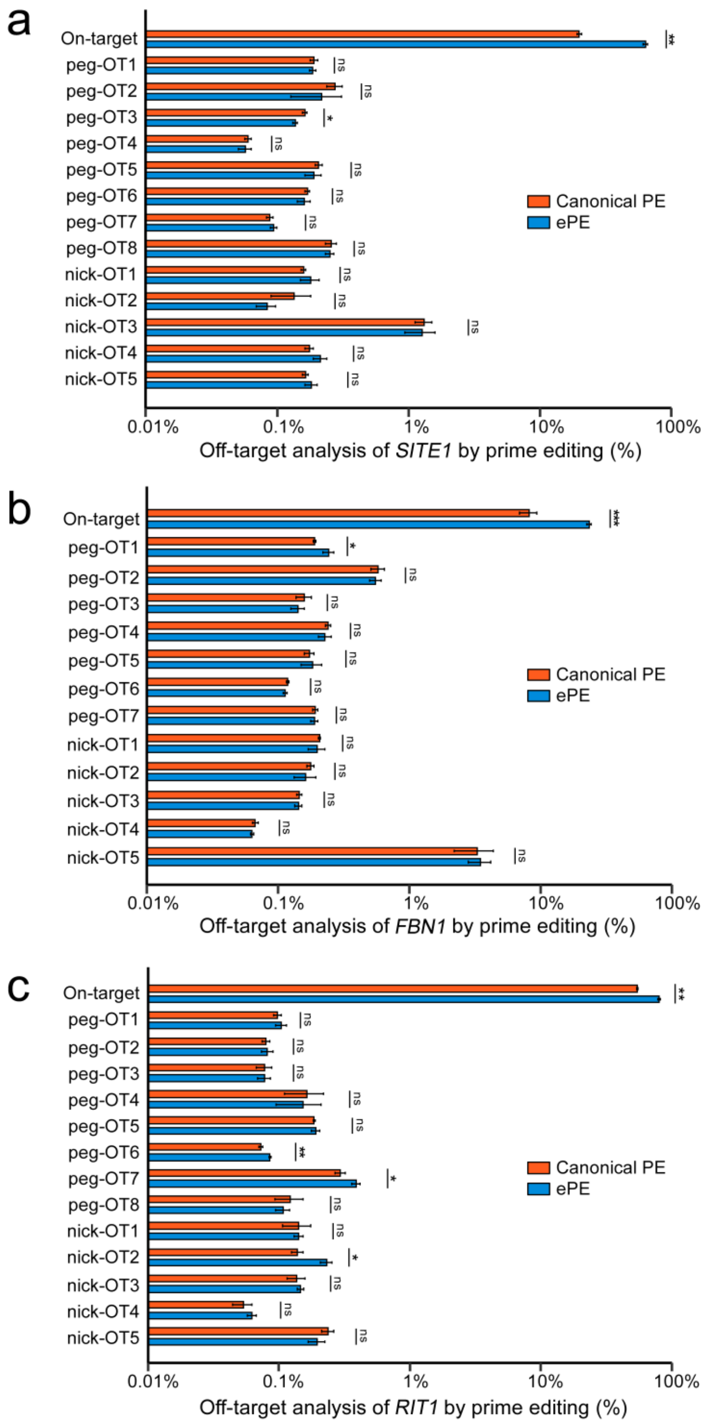

Supplementary information, Fig. S14 Off-target analysis of prime editing induced by canonical PE and ePE.

a. Off-target analysis of prime editing for *SITE1* induced by canonical PE and ePE.

b. Off-target analysis of prime editing for *FBN1* induced by canonical PE and ePE.

c. Off-target analysis of prime editing for *RIT1* induced by canonical PE and ePE. Mean values ± SD, n=3 independent experiments, two-tailed student's *t*-test (*$P < 0.05$, **$P < 0.005$, ***$P < 0.0005$).

Supplementary information, Table S1 Primers used for constructing of pU6-Csy4RS-gRNA plasmids.

NO.	Forward primer
Csy4peg-bone-F	GAGAGGGTCTCAGTTTTAGAGCTAGAAATAGCAAGTTAAAATAAGGCTAGTCCGTTATC
Csy4peg-bone-R	CTCTCGGTCTCACGGTGTTTCGTCCTTTCCAC
scaffold oligo-top	AGAGCTAGAAATAGCAAGTTGAAATAAGGCTAGTCCGTTATCAACTTGAAAAAGTGGCACCGAGTCG
scaffold oligo-bottom	GCACCGACTCGGTGCCACTTTTTCAAGTTGATAACGGACTAGCCTTATTTCAACTTGCTATTTCTAG

Supplementary information, Table S2 pegRNAs and nick sgRNAs used in human cells.

NO.	gene	Spacer sequence	3'-extension sequence	Nick sgRNA sequence	Length of PBS (nt)	Length of RT template (nt)	Type of mutations
ALDOB	*ALDOB*	CCACCCTGTGCTGAGTGTAT	TCTCTCCCTCCAAGACACTCAGCACAGG	TAGCTTCCTATCCAATGCCA	13	15	+2AtoC
FAM171B	*FAM171B*	ATCTGACCTTTTCTCCACAC	TAATTGTTCCGGTCTGGAGAAAAGGTC	TAGACTAACTGTCCCTTTCT	13	14	+1CtoG
FBN1	*FBN1*	GCTTCCTGCTGCTGTTCTCT	AGGCTTTACCCATAGAACAGCAGCAGG	TTTACCCATAGAACAGCAGC	13	14	+2CtoA
KCNA1	*KCNA1*	CCACCTCCCTGGCCATCCTC	AAGCGGATGACCCTAAGGATGGCCAGGGAG	CAAGCGGATGACCCTAAGGA	13	17	+3CtoT
IDS	*IDS*	TTGCCTATAGCCAGTATCCC	TCTGAAGGCCGGAGATACTGGCTATAG	CCTATAGTCTATGGTGCGTA	13	14	+2CtoT
RIT1	*RIT1*	TGCTACAGCAGCTACCAACT	GGAACTCGCCTAGTTGGTAGCTGCTGT	GATTCTGGAACTCGCCTAGT	13	14	+4GtoA
RNF2	*RNF2*	GTCATCTTAGTCATTACCTG	AACGAACATCTCAGGTAATGACTAAGATG	TCAACCATTAAGCAAAACAT	13	16	+6GtoA
SITE1	*ALDOB*	CATACTGTCCTTTGGCCGCC	GCTAACTGCTAGGCGGCCAAAGGACAG	AGGCAGACAGGGTCAAGGTG	13	14	+5GtoA
SITE2	*FAM171B*	TTGTGATCTCTCCATTGAAC	ACAGGAAGACCAGTGCAATGGAGAGATC	GTGTGAGGAGAACAGACAGT	13	15	+1AtoC
MSH2	*MSH2*	ACAAACGTCTCACCCTATGT	ACATTTGGCCCAGATAGGGTGAGACGT	AAAGAAAACAGGGAGAGAAG	13	14	+2GtoC
FTL	*FTL*	CGCGATGATGTGGCTCTGGA	GGCTCACGCCTGCCAGAGCCACATCAT	CTGCCAGAGCCACATCATCG	13	14	+3AtoC
GFAP	*GFAP*	AGCCTGTGTCCATATAAAGG	TTCCAACTCCTCCATTATATGGACACA	CCAGAATCCAATCTCCCTCA	13	14	+1AtoT
SDHB	*SDHB*	CTCAGGTAATCCACCTGCCT	TTGGGAGGCCGAGTCAGGTGGATTACC	ATTTTATAGGCCAGGCGTGG	13	14	+1CtoA
*FAM171B*_+2bp	*FAM171B*	ATCTGACCTTTTCTCCACAC	TAATTGTTCCGGTGGATGGAGAAAAGGTC	TAGACTAACTGTCCCTTTCT	13	16	+2bp
*FBN1*_+2bp	*FBN1*	GCTTCCTGCTGCTGTTCTCT	AGGCTTTACCCAGAGCGAACAGCAGCAGG	TTTACCCATAGAACAGCAGC	13	16	+2bp
*KCNA1*_+2bp	*KCNA1*	CCACCTCCCTGGCCATCCTC	AAGCGGATGACCCTGAGGTGATGGCCAGGGAG	CAAGCGGATGACCCTAAGGA	13	19	+2bp
*RIT1*_+2bp	*RIT1*	TGCTACAGCAGCTACCAACT	GGAACTCGCCCAGTCGTGGTAGCTGCTGT	GATTCTGGAACTCGCCTAGT	13	16	+2bp
*MSH2*_+2bp	*MSH2*	ACAAACGTCTCACCCTATGT	ACATTTGGCCCACAATTAGGGTGAGACGT	AAAGAAAACAGGGAGAGAAG	13	16	+2bp
*SDHB*_+2bp	*SDHB*	CTCAGGTAATCCACCTGCCT	TTGGGAGGCCGAGGCACAGGTGGATTACC	ATTTTATAGGCCAGGCGTGG	13	16	+2bp
*SITE1*_-2bp	*ALDOB*	CATACTGTCCTTTGGCCGCC	GCTAACTGCCAGGGCCAAAGGACAG	AGGCAGACAGGGTCAAGGTG	13	12	-2bp
*RIT1*_-2bp	*RIT1*	TGCTACAGCAGCTACCAACT	GGAACTCGCCCATGGTAGCTGCTGT	GATTCTGGAACTCGCCTAGT	13	12	-2bp
*ALDOB*_-2bp	*ALDOB*	CCACCCTGTGCTGAGTGTAT	TCTCTCCCTCCAACACTCAGCACAGG	TAGCTTCCTATCCAATGCCA	13	13	-2bp
*GFAP*_-2bp	*GFAP*	AGCCTGTGTCCATATAAAGG	TTCCAACTCCTCTTATATGGACACA	CCAGAATCCAATCTCCCTCA	13	12	-2bp
*MSH2*_-2bp	*MSH2*	ACAAACGTCTCACCCTATGT	ACATTTGGCCCATAGGGTGAGACGT	AAAGAAAACAGGGAGAGAAG	13	12	-2bp
*SDHB*_-2bp	*SDHB*	CTCAGGTAATCCACCTGCCT	TTGGGAGGCCGACAGGTGGATTACC	ATTTTATAGGCCAGGCGTGG	13	12	-2bp

Supplementary information, Table S3 pegRNAs used for indel test in human cells.

NO.	Spacer sequence	3'-extension sequence
FBN1-truncated pegRNA	GCTTCCTGCTGCTGTTCTCT	NULL
FBN1-RaPBS_ pegRNA	GCTTCCTGCTGCTGTTCTCT	AGGCTTTACCCATACTAGCGTAGCTAC
FBN1-extended pegRNA	GCTTCCTGCTGCTGTTCTCT	AGGCTTTACCCATAGAACAGCAGCAGGGTTCACTGCCGTATAGGCAG
ALDOB-truncated pegRNA	CCACCCTGTGCTGAGTGTAT	NULL
ALDOB-RaPBS_ pegRNA	CCACCCTGTGCTGAGTGTAT	TCTCTCCCTCCAAGACTAGCGTAGCTAC
ALDOB-extended pegRNA	CCACCCTGTGCTGAGTGTAT	TCTCTCCCTCCAAGACACTCAGCACAGGGTTCACTGCCGTATAGGCAG
SITE1-truncated pegRNA	CATACTGTCCTTTGGCCGCC	NULL
SITE1-RaPBS_ pegRNA	CATACTGTCCTTTGGCCGCC	GCTAACTGCTAGGCCTAGCGTAGCTAC
SITE1-extended pegRNA	CATACTGTCCTTTGGCCGCC	GCTAACTGCTAGGCGGCCAAAGGACAGGTTCACTGCCGTATAGGCAG
FTL-truncated pegRNA	CGCGATGATGTGGCTCTGGA	NULL
FTL-RaPBS_ pegRNA	CGCGATGATGTGGCTCTGGA	GGCTCACGCCTGCCCTAGCGTAGCTAC
FTL-extended pegRNA	CGCGATGATGTGGCTCTGGA	GGCTCACGCCTGCCAGAGCCACATCATGTTCACTGCCGTATAGGCAG

Supplementary information, Table S4 pegRNAs and nick sgRNAs used in mouse N2a cells.

NO.	gene	Spacer sequence	3'-extension sequence	Nick sgRNA sequence	Length of PBS (nt)	Length of RT template (nt)	Type of mutations
Dnmt1	*Dnmt1*	CGGGCTGGAGCTGTTCGCGC	TGCAAGATGCCAGTGCGAACAGCTCCAG	CCGCGCGCGCGAAAAAGCCG	13	15	+2GtoA
Fgf21	*Fgf21*	GTTTTGCCTCACAGGTCTCC	TTTGAGCTCCAGCAGACCTGTGAGGCA	TGAGCTCCAGTAGACCTGTG	13	14	+2CtoG
Ifnar1	*Ifnar1*	AGACGGGAACATGTGGGCAC	AAGGTTTCTCCAGAGCCCACATGTTCCC	AGACTTCTGCCAGATTCGTA	13	15	+2AtoT
Trem2	*Trem2*	TATTATGGAGGCTGGAGTCC	TGGAGTGTACCAGAACTCCAGCCTCCAT	AAGAACACGAATGAGCCAGT	13	15	+2CtoT
Rnf2	*Rnf2*	AGGATGTATTATATTACCTG	AACGAACACCTCCGGTAATATAATACA	TCAACCATTAAGCAAAACAT	13	14	+2TtoG
Tyr	*Tyr*	GAAGTTGCCTGAGCACTGGC	ATAATAGGACCTGCAAGTGCTCAGGCAA	GGACCTCAGTTCCCCTTCAA	13	15	+1GtoT
Fgf5	*Fgf5*	GGCAGCCTGTACTGCAGAGT	AAACCGATGCCCACGCTGCAGTACAGGC	GAGCCATTGACTTTGCCATC	13	15	+1AtoC
Mstn	*Mstn*	CAGAGGGATGACAGCAGTGA	TCCAAAGAGCCATCGCTGCTGTCATCCC	CATGGTAATGATTGTTTCCG	13	15	+1TtoC
Cftr	*CFTR*	GGGGGTTATGTGCTAAGCCA	TGCTTATGGCCATGACTTAGCACATAAC	GGCCTTACTGAGAACTGATC	13	15	+1CtoT
HOXD13	*HOXD13*	GAGGCATACATCTCCATGGA	GACTGGTAGCACTCCATGGAGATGTATG	GATCCTTGGCACAGTACACC	13	15	+6GtoT
SITE3	*HOXD13*	AGTTCATTAACAAGGACAAG	GATCCGCCGCCCCTTGTCCTTGTTAATG	CCTTCGATTCTGAAACCAAA	13	15	+4CtoG
Ar	*Ar*	TCTCACTTGTGGCAGCTGCA	TGAAGAAGACCTTGAAGCTGCCACAAGT	AGGGGAAAATATCAGGAAGT	13	15	+1GtoT
SITE4	*Tyr*	CATTTGCACAGATGAGTACT	GACGACCTCCCAATTACTCATCTGTGCA	CAGGAGGAGAAGAAGGATGC	13	15	+2CtoA

Supplementary information, Table S5 Primers used for cell genomic DNA amplification and targeted deep sequencing.

NO.	Forward primer	Reverse primer	Length of amplicon (nt)
SITE1	CTGAGTGAAGGTTTGACTGG	CTCCTACTAGAAGCACTGGAG	238
FAM171B	GGTAATGAGGAGGCGTATGGGC	GGGCAAGGTCTGCGTAAAGT	213
FBN1	TCGACCTCGAGGAGACAATG	GGGCTGAGAGGACTGATCTTT	252
KCNA1	CACCGAGATAGCTGAGCAGG	GGATGACCCCGATGAAGAGG	206
IDS	ACGTTGAGCTGTGCAGAGAA	TTGAAGCCAACCCACACAGT	235
RIT1	GTATGGAAAGGTAAGGCACTG	CCTACCACTCTTCCCTACACC	237
RNF2	ACGTAGGAATTTTGGTGGGACA	ACGTCTCATATGCCCCTTGG	218
ALDOB	CCTCATTGCCAATGGATCAG	GAGCCCTCACTTTGGGTGTT	235
SITE2	GGCAAACAAGGGAGTAATTC	AGAGAGACGGGAAGCCATTG	262
MSH2	CTCAGCATTCAGTGCTCTCC	TCGCATTTGCACTAGTCCTC	239
FTL	TTTGTGCGGTCGGGTAAACA	CTGCTGGGAGATGTAGTCCAT	269
GFAP	GCCCCTGTGTTTCATTCATG	ACCACCGCTTCACAGCTGTG	250
SDHB	GCCATCGTGCCTGTCTAATT	AGTCGACATATCCCAACATC	260
Dnmt1	TTGCCCTGTGTGGTACATGC	AATATATGCCTCGGCATCGG	251
Fgf21	AGGATGGAACAGTGGTAGGC	CATAGAGAGCTCCATCTGGC	240
Ifnar1	GCCATACTAGTCCACATCTC	CTGGCAAGAGTTCTGGTATC	250
Trem2	GACCTACCTTCAGCAACACT	CTCACAGCTCCTTCAGTGAC	251
Rnf2	GTCTCAGGCTGTGCAGACAA	CAAGACGCAGGACTGTTATG	206
Tyr	CAGCTTTCAGGCAGAGGTTC	CAAGACTCGCTTCTCTGTAC	213
FGF5	TCCTCACCAGTCGCAGCTTC	GCCTGTGGCCCAAAGGAATC	246
MSTN	ATCCTCAGTAAGCTGCGCCT	ACACTAGGACAGCAGTCAGC	250
CFTR	GCCCCTTCTAAGCACAGTGT	TAGATGGGCACTGGGCTCAT	204
Hoxd13	CTACACAAGTCCCTATCAGC	CGTTGCTCCTACCTGGAAAG	209
SITE3	TAAACCAGCCGGACATGTGC	GCCCACATCAGGAGACAGTG	144
Ar	CAGTTTGGACAGTACCAGGG	TCTGCTAGGCAAAAGAGAAGGG	209
SITE4	GAGAACTAACTGGGGATGAG	GAGCATGAAAATGTGGCTGC	216
*FAM171B*_+2bp	GGTAATGAGGAGGCGTATGGGC	GGGCAAGGTCTGCGTAAAGT	213
*FBN1*_+2bp	NNNNNNNNTCGACCTCGAGGAGACAATG	GGGCTGAGAGGACTGATCTTT	260
*KCNA1*_+2bp	CACCGAGATAGCTGAGCAGG	GGATGACCCCGATGAAGAGG	206
*RIT1*_+2bp	NNNNNNNNGTATGGAAAGGTAAGGCACTG	CCTACCACTCTTCCCTACACC	245
SITE2_+2bp	GGCAAACAAGGGAGTAATTC	AGAGAGACGGGAAGCCATTG	262
*MSH2*_+2bp	NNNNNNNNCTCAGCATTCAGTGCTCTCC	TCGCATTTGCACTAGTCCTC	247
*SDHB*_+2bp	NNNNNNNNGCCATCGTGCCTGTCTAATT	AGTCGACATATCCCAACATC	268
*SITE1*_-2bo	CTGAGTGAAGGTTTGACTGG	CTCCTACTAGAAGCACTGGAG	238
*RIT1*_-2bp	NNNNNNNNGTATGGAAAGGTAAGGCACTG	CCTACCACTCTTCCCTACACC	245
*ALDOB*_-2bp	NNNNNNNNCCTCATTGCCAATGGATCAG	GAGCCCTCACTTTGGGTGTT	243
*SITE2*_-2bp	NNNNNNNNGGCAAACAAGGGAGTAATTC	AGAGAGACGGGAAGCCATTG	270
*GFAP*_-2bp	NNNNNNGCCCCTGTGTTTCATTCATG	ACCACCGCTTCACAGCTGTG	256
*MSH2*_-2bp	NNNNNNNNCTCAGCATTCAGTGCTCTCC	TCGCATTTGCACTAGTCCTC	247
*SDHB*_-2bp	NNNNNNNNGCCATCGTGCCTGTCTAATT	AGTCGACATATCCCAACATC	268

Supplementary information, Table S6 Information of predicted off-target sites.

NO.	Chr.	Off-target site	Mis-matches
SITE1-peg-OT1	1	CATAaTGTCCTTTGGaaGCCAGG	3
SITE1-peg-OT2	19	CcTACTGTCCTTTGGgaGCCTGG	3
SITE1-peg-OT3	4	gATACTGTCCTTgGGgCGCCAGG	3
SITE1-peg-OT4	10	CtTtCTGTCCTTTGGCgGCaGGG	4
SITE1-peg-OT5	10	CcTtCTGcCCTTTtGCCGCCTGG	4
SITE1-peg-OT6	1	gATtCTGTCCTgTGGCaGCCTGG	4
SITE1-peg-OT7	11	aATACTGgCCTTTGGCtGgCTGG	4
SITE1-peg-OT8	11	CAcACTGTCCTTTttCCaCCGGG	4
SITE1-nick-OT1	14	AGGCAGcCAGGGTCcAGGTGGGG	2
SITE1-nick-OT2	6	gaGCAGACAGGGTCAAGGTGTGG	2
SITE1-nick-OT3	10	AatCAGcCAGGGTCAAGGTGTGG	3
SITE1-nick-OT4	11	AGGgtGgCAGGGTCAAGGTGTGG	3
SITE1-nick-OT5	1	AGGCAGAgAGGGTgAAtGTGGGG	3
FBN1-peg-OT1	17	GCTTCCTGCTGCTGaTCTCTGGG	1
FBN1-peg-OT2	3	GCTTCCTcCTGgTGTTCTCTTGG	2
FBN1-peg-OT3	10	GtTaCCTGCTGCTGTTtTCTGGG	3
FBN1-peg-OT4	10	aCTTCCTGCTGtTtTTCTCTAGG	3
FBN1-peg-OT5	10	tCTTCCTGCTGCTGTaCTCaTGG	3
FBN1-peg-OT6	10	GCTTCCTcaTGaTGTTCTCTGGG	3
FBN1-peg-OT7	10	GCTTCCTGCTtCTGTcCaCTGGG	3
FBN1-nick-OT1	10	TTTACCCATcGAAtAtCAGCAGG	3
FBN1-nick-OT2	11	TTaACCCAgAGAACAGaAGCAGG	3
FBN1-nick-OT3	15	TTTcCCCATAGAACAcgAGCAGG	3
FBN1-nick-OT4	15	TTTcCCCAcAGAACAGtAGCTGG	3
FBN1-nick-OT5	16	gTTACCCATgGAAaAGCAGCCGG	3
RIT1-peg-OT1	17	TGCTAaAGCAGCTACCtACTTGG	2
RIT1-peg-OT2	12	TGCTACtGCAGCTcCCAgCTGGG	3
RIT1-peg-OT3	12	TtCTgCAGCAGaTACCAACTGGG	3
RIT1-peg-OT4	13	TGCTACAGaAGCagCCAACTAGG	3
RIT1-peg-OT5	14	TGCTtCAGCAGaTACCAgCTGGG	3
RIT1-peg-OT6	15	TGtTcCAGaAGCTACCAACTGGG	3
RIT1-peg-OT7	2	TtCTgCAGCAGaTACCAACTGGG	3
RIT1-peg-OT8	2	TGCaACAGCAGCaACCAcCTGGG	3
RIT1-nick-OT1	4	GATTCTGGAAaTCcCCTtGTTGG	3
RIT1-nick-OT2	11	GcTTCTGGAACTCaCCTccTGGG	4
RIT1-nick-OT3	12	GATTCTGGAtCTtGCCTgGcTGG	4
RIT1-nick-OT4	13	GAcTCTGGgcCTCGCCTtGTAGG	4
RIT1-nick-OT5	13	GATTtTGGtAtTCGCCTAtTTGG	4

Supplementary information, Table S7 Primers used for off-target analysis.

NO.	Forward primer	Reverse primer	Length of amplicon (nt)
SITE1-peg-OT1	CTAGGCACCTTGGAAGCTGC	AGTACCAATGCCACCTCCTCC	257
SITE1-peg-OT2	CCACGGACAGCAACAACTCC	AGACAGTCTCAGAGCTGAGG	214
SITE1-peg-OT3	CACAGGAAAGGGGCGCTGCA	AGTCCACACAGGCAGCAAGC	249
SITE1-peg-OT4	CCGGCCCTGAACATCGTTCT	CTCTCCTGTTTCCCTGACTG	194
SITE1-peg-OT5	TGCGCTCTGCACTCTCTGCT	GGGAAGGTGACCACAGTCAG	243
SITE1-peg-OT6	GGTGGTCCCAAAAGTTACCA	GTCTCCTAGAATTCCCACAGG	218
SITE1-peg-OT7	GCCCTTTCACCCAGAGTCCC	CCCAGCTTGTCACGATCAAGG	221
SITE1-peg-OT8	CCAGCCCACAGAGAATCATG	CTTGGAAAGGCCTGGAGAGG	234
SITE1-nick-OT1	GGCACATCATCCATTCAGGT	CTGGATCTAGCCAGCGAGAC	201
SITE1-nick-OT2	GTGAATGAGACAGCCCAGAG	CTCTGCATGAGGTTCTGTGC	210
SITE1-nick-OT3	GGGAAGATGAAGCTGGGGTA	CCTCCTGCTTCTGGAGACAG	147
SITE1-nick-OT4	CTGCAGATCTTCCTCACCAGG	GTGCCTGCTGATGGGCAGAA	219
SITE1-nick-OT5	GAGAGGAGCAAGCATGGGGA	CCAGCCTTTCCCTACACCTC	232
FBN1-peg-OT1	GGCTACCGTTTTCTGAGCGA	TGGGTACTCAAGCCGGAGAG	246
FBN1-peg-OT2	GTCTGGGAATCGACGCTGAC	TGAATCCGTCTGGTCCTGGAC	259
FBN1-peg-OT3	CCTGCTGGATCTTGGTTCCC	AGAAAGGGAGAACATGCTGC	252
FBN1-peg-OT4	GGATTTCCAACAGCTCAGAAC	CTGTTTCAGGGCTCACTTAG	248
FBN1-peg-OT5	CTAGTCTCTAGCTCTCTCCT	CACCCCCTGAAGAACATTCC	254
FBN1-peg-OT6	GGTCAGGGTTCTTCCAGCCA	GGAGACTTTAGAGAGGGTCC	217
FBN1-peg-OT7	GAGAAGTTGGTCACCTTCCC	TAGGCCAACCAGGTCACCCT	227
FBN1-nick-OT1	TCCAGAGTGACCATAGAATC	CCACCCCAATATTTAGGTGGT	240
FBN1-nick-OT2	AGTGGGTAAACACTACCGGC	AGGTCATGTCTGCCTTGATT	239
FBN1-nick-OT3	CATCACCTTGCACTGGTATA	TAACTCCAGAGTAAACTGAC	231
FBN1-nick-OT4	ATGCTACCTGGGCGGGATTTTG	AGAAAGGAGCAAAGGGGGCT	190
FBN1-nick-OT5	CTGACCGATGGAAAAAAGCC	GCTAGGTTCGTCTGCGTTTCT	196
RIT1-peg-OT1	AGAGACCAAGGAGGGACTGT	TCACCAGAAACGAAACACCAC	221
RIT1-peg-OT2	GGTTTCCAGCAGCCCATTCT	GGAGAAAGAATTCGACCAAG	196
RIT1-peg-OT3	TGGACTTGCCACTGGTAATGT	AAATGTGCTCTTACCCCAGCA	232
RIT1-peg-OT4	GACATTTTCATCAGTGATGCC	GCGGGAGTATCCACAGAATACT	191
RIT1-peg-OT5	TCTTCTCCTACTATGCTATC	TTCCCACATGCCTTGCCTCAT	220
RIT1-peg-OT6	TCAGGATCCTAGCAGGAAGCA	CTCTAATCTTCATATCTGGGG	201
RIT1-peg-OT7	CAGACTTGTGACTGGCATTGC	CCTTTGCTCTCACACCACTAC	233
RIT1-peg-OT8	TCTGCTGCTTTGTTGGAGACAC	AGGTTCCCTTGTCTACTAGC	237
RIT1-nick-OT1	AGCCTCACAATAATGCTGACC	CTTGAGAAACACAGGGTTGT	250
RIT1-nick-OT2	TGAGAAGGCTGGGCAGGGAAAT	CCTTTCCACTTCCAGCCTGA	226
RIT1-nick-OT3	GTTTCCGTGTGAAATGTGTC	CCAAAGACATCTCCCCCTGA	218
RIT1-nick-OT4	AGTTATTTGAGGTCTGGGGG	GCAATGATCAGCACCAAGCT	187
RIT1-nick-OT5	CAGAGTCCAAATCCTTCAGG	CAGCAATTACAGAGGAACAG	240
